# 547 transcriptomes from 44 brain areas reveal features of the aging brain in non-human primates

**DOI:** 10.1186/s13059-019-1866-1

**Published:** 2019-11-28

**Authors:** Ming-Li Li, Shi-Hao Wu, Jin-Jin Zhang, Hang-Yu Tian, Yong Shao, Zheng-Bo Wang, David M. Irwin, Jia-Li Li, Xin-Tian Hu, Dong-Dong Wu

**Affiliations:** 1 State Key Laboratory of Genetic Resources and Evolution, Kunming Institute of Zoology, Chinese Academy of Sciences, Kunming, 650223 Yunnan China; 2 Kunming College of Life Science, University of the Chinese Academy of Sciences, Kunming, 650223 Yunnan China; 3 Key Laboratory of Animal Models and Human Disease Mechanisms of Chinese Academy of Sciences & Yunnan Province, Kunming Institute of Zoology, Chinese Academy of Sciences, Kunming, 650223 Yunnan China; 4 Yunnan Key Laboratory of Primate Biomedicine Research, Institute of Primate Translational Medicine, Kunming University of Science and Technology, Kunming, 650500 Yunnan China; 5 Department of Laboratory Medicine and Pathobiology, University of Toronto, Toronto, ON Canada; 6 Center for Excellence in Brain Science and Intelligence Technology, Chinese Academy of Sciences, Shanghai, 200031 China; 7 National Research Facility for Phenotypic and Genetic Analysis of Model Animals, Kunming Institute of Zoology, Chinese Academy of Sciences, Kunming, 650223 Yunnan China; 8 Center for Excellence in Animal Evolution and Genetics, Chinese Academy of Sciences, Kunming, 650223 Yunnan China

**Keywords:** Brain aging, Rhesus macaques, Transcriptome, Multiple brain regions, *PGLS*

## Abstract

**Background:**

Brain aging is a complex process that depends on the precise regulation of multiple brain regions; however, the underlying molecular mechanisms behind this process remain to be clarified in non-human primates.

**Results:**

Here, we explore non-human primate brain aging using 547 transcriptomes originating from 44 brain areas in rhesus macaques (*Macaca mulatta*). We show that expression connectivity between pairs of cerebral cortex areas as well as expression symmetry between the left and right hemispheres both decrease after aging. Although the aging mechanisms across different brain areas are largely convergent, changes in gene expression and alternative splicing vary at diverse genes, reinforcing the complex multifactorial basis of aging. Through gene co-expression network analysis, we identify nine modules that exhibit gain of connectivity in the aged brain and uncovered a hub gene, PGLS, underlying brain aging. We further confirm the functional significance of PGLS in mice at the gene transcription, molecular, and behavioral levels.

**Conclusions:**

Taken together, our study provides comprehensive transcriptomes on multiple brain regions in non-human primates and provides novel insights into the molecular mechanism of healthy brain aging.

## Background

Aging, an intricate and irreversible process, varies significantly at the individual level, depending on a combination of genetic and environmental factors an individual experiences throughout a lifetime [1–3]. Aging is associated with cognitive decline and memory loss and has been implicated in many neurodegenerative disorders [4–8], thereby posing a major threat to global health. Despite its ubiquity and importance, aging-related alterations have mainly been observed by histology and ethology [9–11], with the underlying molecular mechanisms remaining elusive.

Aging processes are reliant on precise spatiotemporal regulation of the transcriptome, and changes in gene expression have been studied widely in brain aging [12–15]. However, an increasing body of persuasive evidence suggests that aging-related changes depend on the coordination of diversified transcriptional regulation rather than gene expression only [16–20]. One essential mechanism for increasing the spatiotemporal complexity of the transcriptome is alternative splicing, which generates multiple mRNA transcripts from a single gene and affects up to 95% of human multi-exon genes [21]. Moreover, the brain expresses more alternative splicing transcripts than any other tissue [21–23], and dysregulation of alternative splicing may affect healthy brain aging [16].

Currently, emerging evidence from human and animal models suggests that brain aging is regulated by the interaction of multiple brain regions [24], which must work together as a network to control this complex physiological process. Nevertheless, previous research has been limited to only a few brain regions (frontal/prefrontal cortex) [25–27]. Currently, the lack of a genome-wide transcriptional landscape of multiple brain regions limits our understanding of how spatiotemporal orchestration of the transcriptome regulates the process of brain aging.

**Fig. 1:**
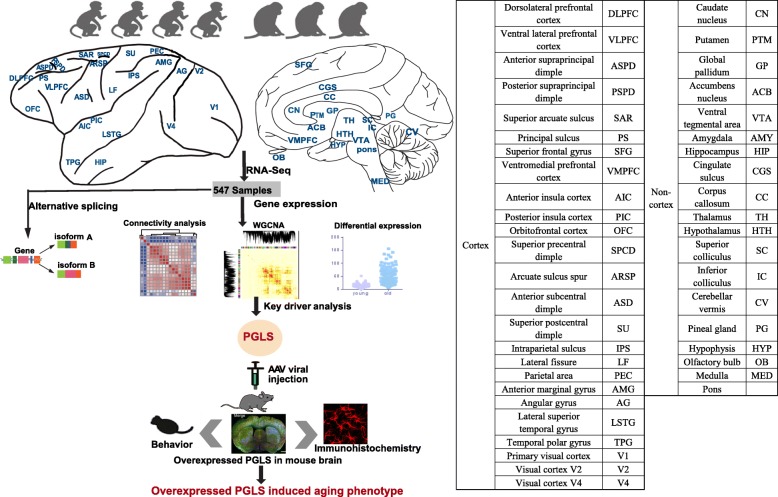
Schematic view of this study. We used 4 young and 3 aged macaques across 44 brain regions to study aging mechanism in NHPs through multifaceted analyses (connectivity analysis, differentially expressed gene analysis, alternative splicing analysis, and network analysis). We further confirmed the role of *PGLS* underlying brain aging in mice. The table on the right shows the ontology and nomenclature of analyzed brain regions

The advent of high-throughput RNA-sequencing (RNA-seq) has allowed for a much more comprehensive exploration of brain aging. In this study, we sequenced the transcriptomes of 44 brain areas from 4 young and 3 aged rhesus macaques (*Macaca mulatta*) (Fig. 1), which are close non-human primate (NHP) relatives of humans (diverging 25 million years ago) [28, 29], to survey transcriptional profile alterations during aging. Through multifaceted analyses of RNA-seq data and integration of gene expression and alternative splicing (Fig. 1), we provide several novel insights into the molecular underpinnings of brain aging. In addition, we discovered a novel hub gene, *PGLS*, underlying brain aging and confirmed its function at the molecular and phenotypic level in mice.

## Results

### Transcriptome profiling across multiple brain areas in rhesus macaques

To investigate the dynamic expression patterns associated with brain aging, we used deep RNA-seq to profile transcriptomes from 590 post-mortem samples isolated from 44 brain areas across the left and right hemispheres in 4 young (5, 6, 6, and 6 years old) and 3 aged (16, 17, and 24 years old) rhesus macaques (Fig. 1). Rhesus macaques reach sexual maturity at 3–4 years and have a typical lifespan of 20 to 30 years in captivity [30]. After rigorous quality control (see the “Methods” section; Additional file 1: Figure S1), 547 samples remained for downstream analyses. RNA-seq data were then normalized, and genes with low expression values were removed to reduce the influence of technical noise. Consequently, 15,531 (61.8%) out of 25,111 genes were detected as having expression signals (an expressed gene was identified as having least 10 fragments in 80% of samples). There were no significant differences in the *RNA* integrity numbers (Mann-Whitney *U* (MWU) test, *p* = 0.723, *N* = 547) or post-mortem intervals (MWU test, *p* = 1, *N* = 547) between samples originating from young and aged groups (Additional file 2: Table S1). Sex, hemisphere, brain region, and individual did not explain a significant amount of expression variation. In contrast, most of the variation in gene expression could be attributed to age (*p* = 0.006; Additional file 2: Table S2), suggesting that age contributes more to global differences in gene expression than any of the other tested variables.

After accounting for the effects of many known biological and technical confounding factors, we performed principal component analysis (PCA) on gene expression in the 547 samples and found that the cortex and non-cortex clearly clustered into 2 separate groups (Additional file 1: Figure S2a). Hierarchical clustering analysis based on inter-array correlation also showed distinct clustering of these two groups (Additional file 1: Figure S2b). Thus, for the following, we studied the cortex and non-cortex in the downstream analyses separately.

### Attenuation of expression connectivity during brain aging in NHPs

**Fig. 2:**
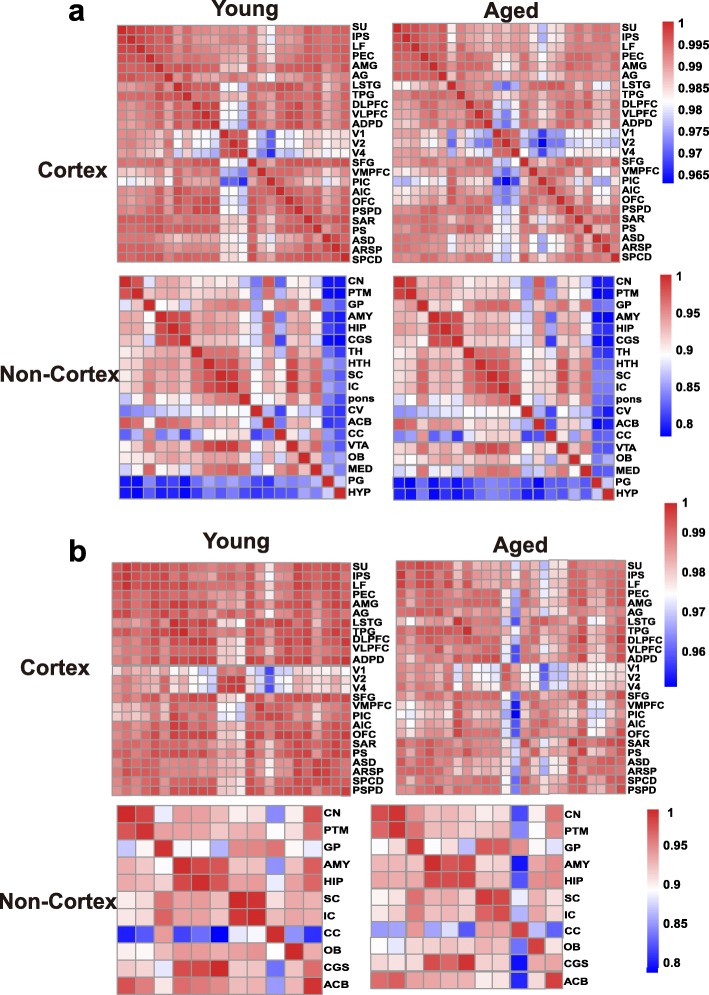
Expression connectivity between pairs of cerebral cortex areas and expression symmetry between the left and right hemispheres decrease after aging. **a** Heat map matrix of pairwise Pearson correlations between cortex regions (top) and between non-cortex areas (bottom) in young and aged macaques. **b** Heatmap matrix of pairwise Pearson correlations between the left and right hemispheres in cortex (top) and non-cortex (bottom) regions in young and aged macaques (columns represent brain areas across the left hemisphere; rows represent brain areas across the right hemisphere)

To assess the changing tendency of transcriptional connectivity among macaque brain regions during aging, we determined the expression correlation between any two brain regions in young and aged groups, respectively. By comparing the correlation matrices at different ages, we found that inter-areal correlations within the cortex decreased after aging (Pearson’s correlation: *p* = 2.00e−09, MWU test), with a less dramatic shift seen in the non-cortex (Pearson’s correlation: *p* = 0.075, MWU test) (Fig. 2a). Moreover, pairwise comparisons of gene expression across all regions of the cortex showed an increase in the number of differentially expressed genes (DEGs) between paired regions during aging (*p* = 0.009, MWU test; Additional file 1: Figure S3a), but no significant change was seen in the non-cortex (*p* = 0.2, MWU test; Additional file 1: Figure S3b). Our results suggest that attenuation of expression connectivity occurs in the cerebral cortex during aging. To further validate this observation, we repeated the correlation analysis using another public age-matched human transcriptome dataset (12–20 to over 60 years old; sampled brain areas can be seen in Additional file 2: Table S3) [31]. Similarly, the expression connectivity among human brain areas was substantially decreased in the cortex (*p* = 1.12e−12, MWU test) but unchanged in the non-cortex (*p* = 0.35, MWU test) after brain aging (Additional file 1: Figure S4), supporting the robustness of our results and indicating conserved and consistent changes in expression connectivity during brain aging in primates.

Additionally, by performing unsupervised hierarchical clustering on the multiple brain areas based on the gene expression signals in the young and aged groups (Additional file 1: Figure S5), we found that the relative relationship between some brain regions was altered during brain aging. For example, the ventromedial prefrontal cortex (VMPFC) clustered closely with the posterior insula cortex (PIC) in young macaques but shifted towards the anterior insula cortex (AIC) and lateral superior temporal gyrus (LSTG) in the aged group, suggesting a dynamic reorganization of transcriptional patterns between brain areas during aging.

The brain hemispheres are known to be anatomically and functionally asymmetric [32, 33]. Thus, to explore changes in expression connectivity between the left and right hemispheres during aging, we determined the correlation matrices of pairwise comparisons between the hemispheres at different ages. Results showed that in the cortex, the correlation coefficient in the young group was significantly higher than that in the aged group (*p* = 0.00011, MWU test), but unchanged in the non-cortex (*p* = 0.7541), suggesting a decreased tendency of transcriptome connectivity between the left and right hemispheres in the cortex during aging (Fig. 2b).

### Transcriptional changes across multiple brain areas during NHP aging

**Fig. 3:**
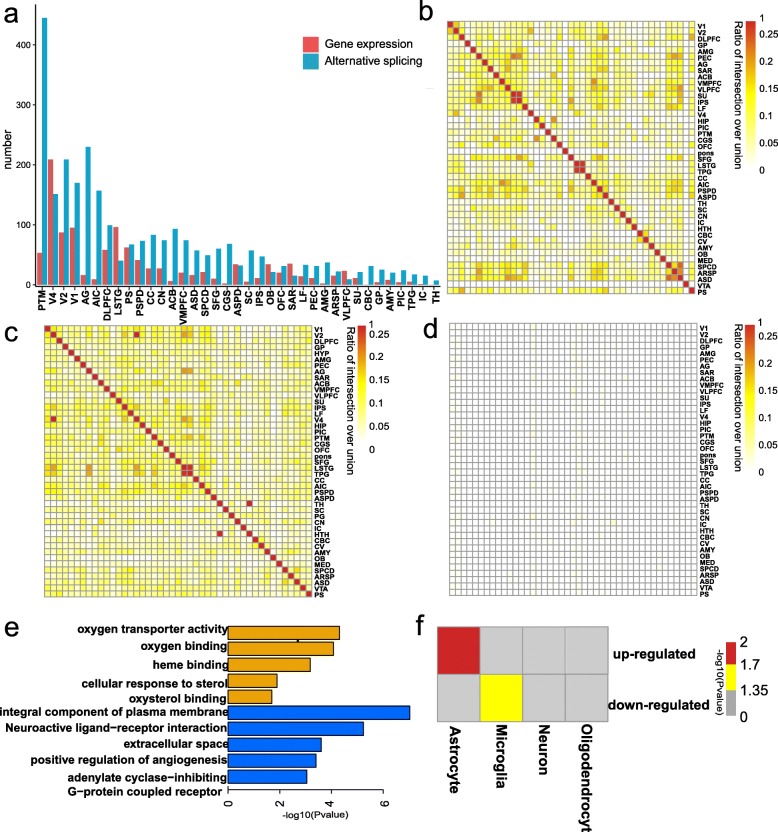
Aging-related transcriptional profile changes. **a** The number of genes with evidence of aging-related gene expression (red) and aging-related alternative splicing (blue) changes. **b** The overlapping rate of DEGs between any two brain regions (the ratio of intersection over union was used to exhibit the overlapping rate). **c** The overlapping rate of genes with DEUs between any two brain regions. **d** The overlapping rate of DEUs and genes with DEGs between any two brain regions. **e** Enriched categories for upregulated (top) and downregulated (bottom) DEGs in aged macaques. **f** Matrix summary of enrichment in oligodendrocyte, neuron, microglia, endothelial, or astrocyte genes in upregulated and downregulated DEGs of aged macaques

We next investigated the broad patterns of aging-related transcriptome changes in each brain area by integration of gene expression (*p* < 0.05, fold change [FC] > 1.5) and alternative splicing (*p* < 0.01). Results showed a positive correlation between the number of DEGs and genes with differential exon usage genes (DEUs) across brain regions (Pearson’s *r*^2^ = 0.39, *p* = 0.018; Additional file 1: Figure S6). In addition, changes in gene expression and alternative splicing were widespread in all brain regions, although the changes were not uniform (Fig. 3a). Focusing on the 37 brain regions with similar sample sizes, the visual cortex V4 area was the most prioritized region exhibiting changes in gene expression (Fig. 3a). In terms of alternative splicing, the putamen (PTM) was the most pronounced region showing aging-related changes (Fig. 3a). Although the putamen plays an important role in cognitive ability [34–36], few studies have focused on its role in brain aging.

A significant overlap of DEGs was found across brain areas (Fig. 3b; Additional file 2: Table S4), and genes with DEUs were also widely shared among different brain areas (Fig. 3c; Additional file 2: Table S5). These findings suggest that although the degree of aging-related change across brain areas was diversified (Fig. 3a), aging mechanisms among different brain regions were largely convergent. However, we found a rare overlap between DEGs and genes with DEUs across brain regions (Fig. 3d; Additional file 2: Table S6). Gene enrichment analyses also indicated that DEGs and genes with DEUs were enriched in different categories (Additional file 1: Figure S7). Our results suggest that gene expression and alternative splicing likely regulate brain aging in distinct manners.

In consideration of the convergent mechanisms among different brain areas during aging described above, we next investigated aging-related gene expression changes in the whole cortex and whole non-cortex. In the cortex, we identified 432 DEGs (157 upregulated, 275 downregulated) (*p* < 0.05, fold change [FC] > 1.5) accounting for 2.8% (432/15,220) of all expressed genes. In the non-cortex, we identified 268 DEGs (86 upregulated, 182 downregulated) equating to 1.7% (268/15,531) of expressed genes. The decreased number of DEGs in the non-cortex (*p* = 6.186e−10, chi-squared test) is consistent with recent functional magnetic resonance imaging (fMRI) research, which showed that aging induced more dramatic changes in the cortex than in the non-cortex [37]. However, a highly significant overlap in DEGs was found between the cortex and non-cortex (*p* = 1.5e−224, Fisher’s exact test; Additional file 1: Figure S8), corroborating the conclusion that aging-related gene expression changes are largely convergent among the different regions.

The NDRG family member 4 (*NDRG4*) gene, highlighted in our analysis, is reportedly implicated in Alzheimer’s disease (AD) [38]. Our results showed that the expression level of *NDRG4* significantly decreased during aging (1.5-fold change, unpaired *t* test, *p* = 9.29e−07), which agrees with previous studies showing that *NDRG4* mRNA expression is lower in the brains of patients with AD [39]. We speculate that *NDRG4* plays an important role in regulating brain aging. Another interesting gene identified in our analysis was cytochrome c oxidase III, mitochondrial (*MT-CO3*), which was upregulated in the aged brains (2.3-fold change, unpaired *t* test, *p* = 9.53e-39). The main function of this gene is to regulate cytochrome-c oxidase activity and respiratory electron transfer activity [40]. *MT-CO3* has also been implicated in AD, Huntington’s disease (HD), and Parkinson’s disease (PD) [41, 42]; however, no previous study has reported an association between *MT-CO3* and brain aging. Our study suggests that *MT-CO3* is likely involved in brain aging.

To further characterize the observed DEG patterns, we examined the enrichment of cell type-associated genes and gene ontologies for the significantly up- and downregulated genes in the aged group. Genes with upregulated expression were predominantly enriched in astrocytes (Fig. 3f; Additional file 1: Figure S9). Gene Ontology (GO) enrichment analysis indicated that they were associated with oxygen transporter activity (Fig. 3e; Additional file 2: Table S7). In contrast, downregulated genes were enriched in microglia (Fig. 3f; Additional file 1: Figure S9) and were involved in neuroactive ligand-receptor interaction and angiogenesis pathways (Fig. 3e; Additional file 2: Table S8). The transcriptional patterns of identified DEGs are consistent with previous studies [4, 43, 44].

### Gene co-expression analysis reveals network reorganization in aged brains

**Fig. 4:**
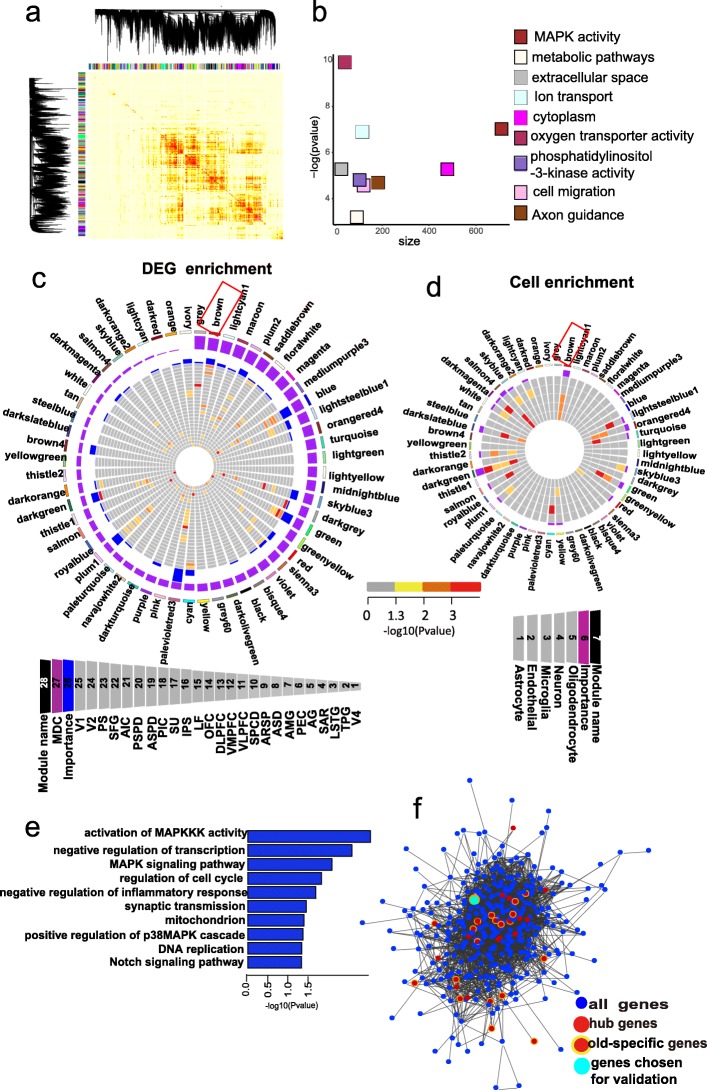
Weighted gene co-expression network analysis (WGCNA). **a** In total, 56 modules were identified by WGCNA. **b** Significant (FET *p* value after correcting for number of modules and functional categories/pathways tested) enrichment of functional categories in modules with gains of connectivity. *Y*-axis represents – log (*p* value) of enrichment; *x*-axis denotes number of genes per module. **c** Circos plots displaying degree of enrichment for DEGs in aged-brain modules. Outermost rectangle is an arbitrary color for module name, followed by MDC score and then by importance (a measure considering degree of enrichment for DEGs across brain regions). Innermost concentric circles represent degree to which DEGs are contained within a given module for each brain region. **d** Circos plots displaying degree of enrichment for cell types in aged-brain modules. Outermost rectangle is an arbitrary color for module name, followed by importance (a measure considering degree of enrichment for cell types). Innermost concentric circles represent enrichment for genes with fivefold higher expression in oligodendrocyte, neuron, microglia, endothelial, or astrocyte cell types (Zhang et al. [94]) in aged-brain modules. **e** Functional enrichment of genes in brown module. **f** Network plot of hub genes identified within brown module. Blue nodes indicate all genes. Red nodes indicate hub genes. Yellow halos indicate aged-specific hub genes. Cyan node indicates gene *PGLS* for functional validation. Edges reflect significant interactions between genes based on mutual information

To gain further insight into the molecular mechanisms involved in brain aging, we applied weighted gene co-expression network analysis (WGCNA) to profile the aged-brain transcriptome into a higher order [45–47]. A total of 56 modules ranging in size from 24 to 1844 gene members were identified (Fig. 4a). Remarkably, we observed significant evidence that 46 of the 56 modules were preserved in an independently published transcriptome dataset, which contained frontal cortex expression data from 478 people collected to study aging [25] (Additional file 1: Figure S10), thus suggesting robustness of the co-expression networks constructed here.

We next used modular differential connectivity (MDC), i.e., the ratio of the average connectivity for any pair of module-sharing genes in the aged group compared to that for the same genes in the young group, to quantify the network reorganization across young and aged groups [48]. Among the 56 modules, 9 (16.1%) showed gain of connectivity, none showed loss of connectivity, and 47 (83.9%) showed no change in connectivity in the aged group compared to the young group (Additional file 2: Table S9). The modules showing a gain of connectivity in the aged brain contained diverse functional categories (Fig. 4b; Additional file 2: Table S9), including “MAPK activity” (brown, *p* = 8.82E−4), “metabolic pathways” (floral white, *p* = 0.04), “oxygen transporter activity” (maroon, *p* = 4.92E−5), “phosphatidylinositol-3-kinase activity” (medium purple 3, *p* = 0.001), “Axon guidance” (saddle brown, *p* = 0.009), and “extracellular space” (gray, *p* = 0.005). Many of these functional categories have previously been implicated in brain aging [49–53], reinforcing the conclusion that complex multifactorial mechanisms underlie brain aging.

We ranked the modules based on the degree of DEG enrichment across multiple cortex regions. Of the 56 modules, 34 were enriched in DEGs in at least 1 brain region (Fig. 4c). The brown module was of particular interest as it was highly enriched in DEGs across brain regions (Fig. 4c) and showed gain of connectivity in the aged network (Additional file 2: Table S9). Furthermore, genes in the brown module were enriched in microglia cells and astrocytes (Fig. 4d). To further explore the profile of the brown module, we performed GO enrichment analysis and found that the most prominent functions were related to activity of mitogen-activated protein kinases (MAPKs) (Fig. 4e). MAPKs are serine-threonine kinases that mediate intracellular signaling and play an important role in regulating aging [54–56], with deviation from strict control of the MAPK signaling pathways implicated in many human neurodegenerative diseases, including AD and PD [57, 58].

Further, we reconstructed the network structure of genes within the brown module solely on the basis of their connectivity and identified the so-called hub genes and aged-specific hub genes. Hub genes are genes with the highest degree of connectivity within a module and are expected to control the expression of many other module members [45]. Aged-specific hub genes were found in the aged group, but not in the young group, and thus may be especially important in generating gene co-expression networks unique to senility. We identified 48 hub genes in the brown module, 20 of which were aged-specific hub genes (Fig. 4f; Additional file 2: Table S10).

### Function of hub gene *PGLS* in brain aging

We validated our bioinformatic predictions by focusing on *PGLS*, a highly connected aged-specific hub gene within the brown module and found to be upregulated in the aged macaque brain (*p* = 0.04), as described in our DEG analysis above. Upregulation of *PGLS* in the aged macaque brain was also confirmed by real-time quantitative polymerase chain reaction (qRT-PCR) (*p* = 0.029; Additional file 1: Figure S11). *PGLS* encodes 6-phosphogluconolactonase, which catalyzes the hydrolysis of 6-phosphogluconolactone in the second step of the pentose phosphate pathway [59]. Although little is known about the function of *PGLS* in brain aging, the pentose phosphate pathway is reported to be broadly involved in the aging process [60–62].

**Fig. 5:**
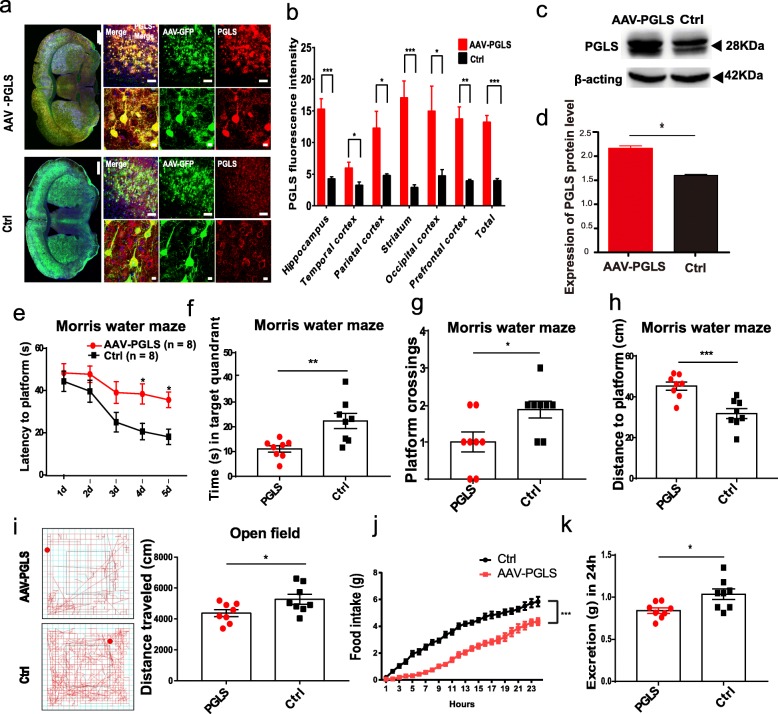
Overexpression of *PGLS* gene in mice causes aging phenotypes. **a** Immunostaining of coronal sections of brains from AAV-PGLS and control (Ctrl) mice for GFP (green) and PGLS (red). Scale bars: large = 1 mm, middle = 100 μm, and small = 10 μm. **b** Fluorescence intensity of PGLS protein detected by anti-PGLS antibody obtained from GFP-positive cells was quantified and averaged (unpaired *t* test with Welch’s correction: hippocampus *p* = 0.0002, temporal lobe *p* = 0.022, parietal lobe *p* = 0.0259, striatum *p* = 0.001, occipital *p* = 0.0366, prefrontal cortex *p* = 0.0011, and total *p* < 0.0001). **c** Representative immunoblots of PGLS in brains from AAV-PGLS and Ctrl mice at 12 months of age. **d** Protein expression level of PGLS in brains from AAV-PGLS and Ctrl mice (unpaired *t* test with Welch’s correction, *p* = 0.0123). **e** Latencies (second) during training in Morris water maze of PGLS with Ctrl (*n* = 8 mice, two-way ANOVA with Bonferroni’s multiple comparison test.). **f** Time (second) spent in goal quadrant during Morris water maze probe trial (*n* = 8, unpaired *t* test with Welch’s correction, *t* = 3.364, *p* = 0.0078). **g** Number of platform crossings during Morris water maze probe trial (*n* = 8, unpaired *t* test, *t* = 2.497, *p* = 0.0256). **h** Swimming distance (cm) to platform during Morris water maze probe trial (*n* = 8, unpaired *t* test, *t* = 4.244, *p* = 0.0008). **i** Examples of results obtained from open field test trace image (left). Total distance traveled (*n* = 8, unpaired *t* test, *t* = 2.296, *p* = 0.0376) in open field test during a 20-min period (right). **j** Cumulative food intake over a 24-h period (*n* = 8, repeated-measure ANOVA, *F* = 3.169, ***p < 0.0001, ηp^2^ = 0.303). **k** Total excretions (g) in 24 h (*n* = 8, unpaired *t* test, *t* = 2.747, *p* = 0.0157)

*PGLS* is a conserved gene among mammals and expressed endogenously in both macaques and mice (Additional file 1: Figure S12) [63, 64]. To address the functional role of higher *PGLS* levels in brain aging, an engineered adeno-associated virus (AAV) combined with a green fluorescent protein (GFP) tag was used to overexpress *PGLS* in the central and peripheral nervous systems of 6-month-old C57BL/6 J male mice by caudal intravenous injection [65, 66], abbreviated here as AAV-PGLS mice. As a control group, the same AAV vector containing the GFP tag was injected into the remaining mice (Ctrl mice). As expected, both immunohistochemical and Western blot analyses showed that *PGLS* was significantly overexpressed in the whole brain (including the cortex and non-cortex) of AAV-PGLS mice until the age of 12 months (Fig. 5a–d; Additional file 1: Figure S13). At the cell level, in addition to microglial cells, both neurons and astrocyte cells were transduced (Additional file 1: Figure S14).

To examine whether overexpression of *PGLS* induced aging-related behaviors, we tested AAV-PGLS (*n* = 8) and Ctrl (n = 8) mice with the Morris water maze (MWM) task. Before AAV injection, there were no significant differences between the two groups of mice in the MWM task (6 months old) (Additional file 1: Figure S15). However, 6 months after virus injection, the AAV-PGLS mice (12 months old) displayed an impairment in learning the new platform location during the acquisition phase of the MWM relative to Ctrl mice (12 months old) (Fig. 5e). Memory dysfunction in mice overexpressing *PGLS* was also observed during the probe trial. Compared to Ctrl mice, the AAV-PGLS mice spent less time in the target quadrant (*p* = 0.0078; Fig. 5f) and crossed the platform location less frequently (*p* = 0.0256; Fig. 5g). The swimming distance to reach the platform location was also longer for AAV-PGLS mice (*p* = 0.0008; Fig. 5h). Thus, the MWM results indicate that overexpression of *PGLS* induced memory impairment.

Additionally, open field tests verified that the distance traveled by AAV-PGLS mice was shorter than that by Ctrl mice (*p* = 0.037; Fig. 5i), suggesting that overexpression of *PGLS* also caused impairment in locomotor activity [67]. However, compared to the Ctrl, AAV-PGLS mice displayed normal motor coordination and balance with the rotarod test (Additional file 1: Figure S16).

As aging usually accompanies a physiological decrease in food intake [68–71], we also tested food intake in the AAV-PGLS and Ctrl mice. Results showed that AAV-PGLS mice exhibited decreased food intake and fecal output compared to the Ctrl mice (Fig. 5j, k), indicating that overexpression of *PGLS* resulted in decreased appetite, a key indicator of aging [68].

**Fig. 6:**
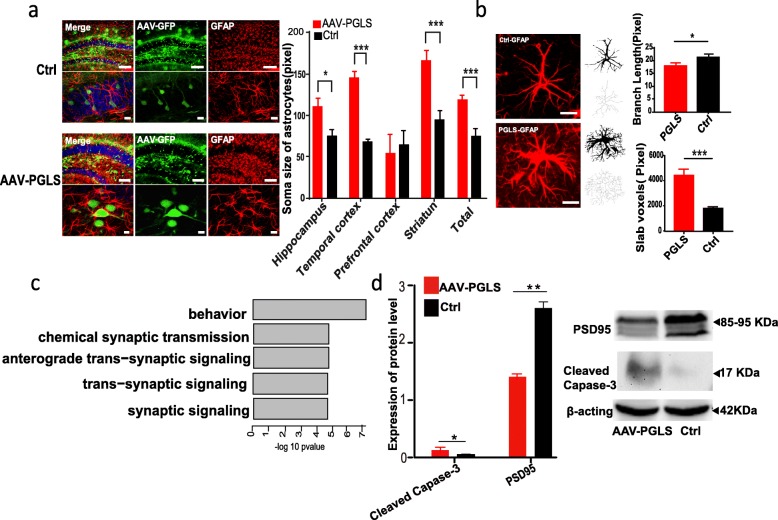
Molecular functional study of *PGLS*. **a** Soma size of GFAP-positive astrocytes was substantially larger in AAV-PGLS mice than in control (Ctrl) mice in most brain regions (unpaired test or unpaired *t* test with Welch’s correction: hippocampus *p* = 0.0158, temporal cortex *p* < 0.0001, prefrontal cortex *p* = 0.7358, stratum *p* = 0.0008, and total *p* = 0.001; scale bars: large = 100 μm and small = 10 μm). **b** Example photomicrograph of fluorescent IHC and cropped cell with skeletonized image. AAV-PGLS group (*n* = 10 cells) had significantly shorter branch length and more slab voxels than AAV-control group (*n* = 10 cells) (branch length: unpaired *t* test with Welch’s correction *t* = 2.709, *p* = 0.019; slab voxels: unpaired *t* test with Welch’s correction *t* = 5.17, *p* = 0.0004). **c** Functional enrichment of differentially expressed genes after overexpression of *PGLS*. **d** Representative immunoblots of PSD95 and caspase-3 in brains from AAV-PGLS and Ctrl mice at 12 months of age (*p* = 0.0094 for PSD95; *p* = 0.0383 for caspase-3; unpaired *t* test with Welch’s correction)

Alterations in astrocyte morphology are an important hallmark of brain aging [72, 73]. Thus, we investigated changes in the microscopic morphology of astrocytes after *PGLS* overexpression through in vitro and in vivo tests. When upregulated *PGLS* astrocyte cell systems were maintained in culture, they showed a senescence-related feature [74], i.e., an increase in nuclear size (*p* = 2.2e−16) comparable to that observed in Ctrl cells undergoing replicative senescence (Additional file 1: Figure S17). We also conducted immunohistochemical mapping of 12-month-old mouse brains to observe the microscopic morphology of astrocytes and found that the soma size of the glial fibrillary acidic protein (GFAP)-positive astrocytes was substantially larger in AAV-PGLS mice than in Ctrl mice (Fig. 6a). In addition, the astrocytes in AAV-PGLS mice exhibited a stubbier morphology compared to those in Ctrl mice (Fig. 6b). Together, the characteristics of AAV-PGLS astrocytes are consistent with their previously reported senescence phenotype [73, 75], thereby highlighting the physiological relevance of *PGLS* in brain aging.

To further investigate the mechanisms linking *PGLS* to brain aging, we performed RNA-seq on the brains of 12-month-old AAV-PGLS and age-matched Ctrl mice. Differential expression analysis identified 73 DEGs induced by *PGLS* overexpression (*p* < 0.05). Gene enrichment analysis indicated that the DEGs were mainly involved in synapse-related pathways (Fig. 6c). Thus, we measured the level of PSD95, a key synaptic protein, in the brains of Ctrl mice and those overexpressing *PGLS*. As expected, Western blot analyses of anti-PSD95 showed a significant decrease in protein levels in the AAV-PGLS mice compared to the Ctrl mice (*p* = 0.0094, Fig. 6d), indicating that *PGLS* overexpression induced loss of synapses.

Brain aging is also associated with a decrease in the number of cells, with apoptosis reported to be a major factor contributing to the loss of cells with age [76, 77]. Thus, we compared the activity of caspase-3, a key executor of apoptosis [77], in AAV-PGLS and Ctrl mice. Western blot analysis showed that cleaved caspase-3 activity was significantly increased after *PGLS* overexpression (*p* = 0.0383, Fig. 6d), indicating that overexpression of *PGLS* induced elevated rates of apoptosis, thus conforming to the characteristics of brain aging.

## Discussion

We applied large-scale RNA-seq on multiple areas of the macaque brain to uncover novel molecular mechanisms and biomarkers related to aging in NHPs. Our results should deepen our understanding of the process of brain aging.

Based on comparison of gene expression profiles between young and old macaques, we found a decreased tendency of expression correlation among multiple brain areas after aging. Furthermore, combined with previous conclusions that expression correlations between major brain areas increase during human early brain development [28], we hypothesized that connectivity within the brain exhibits a “mountain-like” pattern across a lifespan, i.e., connectivity first increases during early brain development and then decreases with aging. In addition, the changes of expression profile after aging would reveal to some extent declines of brain functions, e.g., cognitive function. For example, it is well known that multiple brain areas must work together to accomplish complex cognitive functions [76]. Our results indicated that the connectivity between brain regions decreased after aging, which was consistent with loss of cognitive function in the process of brain aging.

We also found that changes in gene expression and alternative splicing were widespread across all brain areas, although variability existed in the number of genes that changed with age in the different brain regions, thus suggesting that the degree of aging in different brain areas may differ. However, despite this, the aging mechanism among different brain regions was largely convergent. Additionally, we found that different forms of transcription regulation (e.g., gene expression and alternative splicing) acted on brain aging in distinct manners, thereby reinforcing the complex multifactorial basis underlying the aging process.

Among the 44 brain regions analyzed, the putamen was highlighted as showing dramatic transcriptional changes during aging. However, few studies have focused on this region previously. The putamen is located at the base of the forebrain and together with the caudate nucleus forms the dorsal striatum [78]. Furthermore, it is reported to play an important role in cognitive functions, including learning, decision making, and motor behaviors [79–81], and is implicated in various neurological diseases, such as PD, AD, and HD [35, 78]. Our results suggest that the putamen should receive greater attention in future research, diagnosis, and treatment of brain aging. In addition to the putamen, other brain regions account for special functions and tasks, like learning, memory, and language. The large-scale transcriptome data obtained from the multiple brain regions in this study should provide insight into the functional changes that occur after aging for different regions based on changes in expression profiles.

Prior studies on the transcriptional mechanisms of brain aging have focused primarily on identifying individual candidate genes or profiling whole transcriptomes within single brain regions in isolation. In the present study, we applied a network-based approach to reveal inter-regional co-regulation gene signatures associated with brain aging, and identified multiple modules showing increased connectively in the aged brain. These modules were enriched in a number of different pathways, highlighting the intricate mechanisms underlying brain aging. By analyzing the key driver genes within these modules, we provided compelling evidence to support *PGLS* as a key hub gene in brain aging. Upregulation of *PGLS* in mice resulted in impaired memory and locomotor activity, as well as decreased food intake. In addition, the morphology of astrocytes exhibited a senescent phenotype after *PGLS* overexpression. We found that the consequence of *PGLS* overexpression in brain aging is likely through synapse loss. We propose that *PGLS* should be regarded as a novel biomarker of brain aging in future research. We confirmed the function of *PGLS* in mice; however, future experiments on *PGLS* in primates are necessary to further confirm the functions of this gene.

## Conclusions

This study provides novel insight into the molecular mechanism of healthy brain aging based on a comprehensive transcriptome map across multiple brain regions and confirmed a novel gene (*PGLS*) related to brain aging in mice, which will be an important resource to the neuroscientific community.

## Methods

### Sample preparation

The brains of four young (5, 6, 6, and 6 years old) and three aged (16, 17, and 24 years old) rhesus macaques with no previously reported neuropsychiatric disorders were obtained from the Kunming Primate Research Center, Chinese Academy of Sciences(AAALAC accredited).

According to a widely used macaque brain atlas (http://www.brainmaps.org), tissues spanning 44 anatomically distinct regions were selected and collected by a skilled technician with over a decade of experience, and he is also an operator of brain dissection in another studies [82, 83]. We applied a list of previously published gene markers specific to human brain regions to validate the accuracy of the brain dissection [84] (Additional file 1: Figure S18). Brain dissection of the seven macaques was performed from fresh specimens by the same person to ensure consistency in sampling between specimens. Surgical instruments were sterilized in advance, and surgical scissors and tweezers were only used one time for each sample to avoid cross-contamination. Only the central portion of each brain region was sampled. Each sample consisted of 100 mg of dissected tissue. All collected samples were washed with RNAlater solution (AM7021, Ambion, USA) and placed in freezing tubes for storage at liquid nitrogen temperature.

Total RNA was extracted using an RNeasy Plus Universal Kit (Qiagen). Quality and quantity measurements of the extracted RNA were performed using NanoDrop (Thermo Fisher Scientific) and a Qubit Fluorometer (Thermo Fisher Scientific), respectively, and RNA Integrity Numbers (RIN) were determined using a Bioanalyzer RNA 6000 Nano Kit (Agilent, USA). All procedures were approved by the Institutional Animal Care and Use Committee (IACUC) at the Kunming Institute of Zoology (approval number: SMKX2017021).

### RNA-sequencing

A paired-end sequencing library was constructed from poly (A)^+^ RNA, as described in the Illumina manual, and sequenced on the Illumina Hiseq 2000 sequencing platform. For each sample, 5 G of data were generated by RNA-seq. Sequencing data were deposited in the Genome Sequence Archive database (http://gsa.big.ac.cn/) under accession ID CRA000336 for 590 transcriptomes in the macaque brain.

### Read alignment and quality control

We acquired 590 transcriptomes across 44 brain regions from 4 young and 3 aged macaques. First, quality control (QC) of RNA was performed based on the RIN (see Additional file 2: Table S1), with 1 sample excluded after failing our cutoff of RIN ≤ 5. We next use Btrim64 to trim reads to obtain high-quality reads [85]. The paired-end reads were mapped to the macaque reference genome using Tophat2 [86]. The *rmdup* command in Samtools was used to remove PCR duplication of bam files [87], with the *SortSam* command in PicardTools (http://broadinstitute.github.io/picard/) then used to sort bam files.

After read alignment, QC analysis was performed using PicardTools v1.100 (commands *ReorderSam*, *CollectAlignmnetSummaryMetrics*, *CollectRnaSeqMetrics*, *CollectGcBiasMetrics*). Sequencing metrics were used to remove samples with poor sequence quality based on the following sequencing metrics: %Total Reads, %High-quality Aligned Reads, %mRNA Bases, %Intergenic Bases, Median 5′ to 3′ Bias, GC Dropout, and AT dropout (Additional file 2: Table S1). To detect outliers, a quality *z*-score was calculated for each metric, and samples with low quality (Z > 2 for %Intergenic Bases, GC Dropout, or AT Dropout and Z < − 2 for %Total Reads, %High-quality Aligned Reads, %mRNA Bases, or Median 5′ to 3′ Bias) in this matrix were identified as outlier values, and any sample with greater than one outlier value was removed due to sequencing quality concerns. QC analysis was performed for the 590 initial samples, with 43 samples (7%) thus removed. The remaining 547 samples were used for downstream analysis (Additional file 1: Table S1).

### Quantification and adjustment of gene expression

Gene expression levels were quantified for samples passing QC using HTSeq (v.0.6.1) [88]. Genes were retained if expressed in 80% of samples, with HTSeq quantification of 10 counts (thus removing genes supported by only a few reads) within all cortex and non-cortex samples separately. We used cqn software to adjust GC content according to the GC content results from the *CollectGcBiasMetrics* command in PicardTools as well as sequencing depth according to read length [89].

After that, we adjusted the data for covariates, including sex, batch, and sequencing quality metrics (Additional file 2: Table S1). Given the large number of sequencing quality features, we performed principal component analysis (PCA) on these data and found that the first two PCs on the unstandardized features explained nearly 99% of the variance. Consequently, we opted to use two sequencing surrogate variables (seqSV1 and seqSV2) as covariates. We applied a linear model to remove the confounding factors:

$$ \mathrm{adjusted}\_\mathrm{value}=\mathrm{original}\_\mathrm{value}-\mathrm{batch}\times \mathrm{beta}.\mathrm{batch}-\mathrm{sex}\times \mathrm{beta}.\mathrm{sex}-\mathrm{seqSV}1\times \mathrm{beta}.\mathrm{seqSV}1-\mathrm{seqSV}1\times \mathrm{beta}.\mathrm{seqSV}2. $$

### Gene clustering analysis

Based on the expression values, PCA from the prcomp R package (https://www.r-project.org/) was used to visualize the relatedness of all 547 RNA-seq samples. We also used agglomerative hierarchical clustering in the flashClust R package [90] to perform clustering analysis.

### Transcriptional connectivity analysis in young and aged macaques

Pairwise Pearson and Spearman correlation coefficients of gene expression values between any two brain regions and between the left and right hemispheres were calculated in the young and aged macaques using R (https://www.r-project.org/). The Mann-Whitney *U* (*MWU*) test was used to compute the statistical significance of the correlations between young and aged groups. The *MWU test* was executed using the R function *wilcox.test()*, the command *correct = TRUE* was used to adjust *p* values, and a continuity correction was applied to the normal approximation for the *p* value.

### DEG analysis between young and aged macaques

Pairwise differential expression between the young and aged macaques was investigated with the DESeq2 R package [91]. A nominal significance threshold of *p* < 0.05 and fold change (FC) > 1.5 was used to identify DEGs. The *p* value was adjusted for multiple testing using Benjamini-Hochberg to estimate the false discovery rate (FDR). Two online resources were utilized, i.e., DAVID (https://david.ncifcrf.gov/) and g:Profiler (https://biit.cs.ut.ee/gprofiler/), to assess the enrichment of functional categories (GO and KEGG) of the DEGs [92, 93]. The *p* value was adjusted for multiple testing using Benjamini-Hochberg to estimate the false discovery rate (FDR). To assess cell-type specificity in the upregulated or downregulated genes in the aged group, we used genes expressed at least fivefold higher in one cell type than all other cell types (neuron, microglia, astrocyte, oligodendrocyte, and endothelial) from brain-based RNA expression data [94].

### Alternative splicing analysis in multiple brain regions during aging

The DEXSeq R-package [95] was used to test for differential exon usage (DEU) with default parameters. The *p* value significance level was set to 0.01 for detecting significant DEUs and was adjusted for multiple testing using Benjamini-Hochberg to estimate the FDR.

### Construction of gene co-expression modules for aged brains

We used the aged macaque gene expression data to construct multi-tissue co-expression networks that simultaneously captured intra- and inter-tissue gene-gene interactions [45, 48]. Before identifying co-expressed gene modules, we used the linear regression model to correct the effect of brain region covariates on expression values. To quantify the differences in transcription network organization between the young and aged samples, we employed modular differential connectivity (MDC) metrics [48, 96]. In brief, MDC represents the ratios of the connectivity of all gene pairs in a module from the aged samples to that of the same gene pairs from the young samples, with MDC > 0 indicating a gain of connectivity or enhanced co-regulation between genes in aged samples, and MDC < 0 indicating a loss of connectivity or reduced co-regulation between genes in the aged group. As a result, among the 56 aged modules, 9 showed gain of connectivity, none showed loss of connectivity, and 47 showed no change in connectivity compared to the young group.

To identify key regulator (driver) genes in the brown module, we applied key driver analysis to the module-based unweighted co-expression networks derived from ARACNE [97]. ARACNE first identified significant interactions between genes in the brown module based on their mutual information and then removed indirect interactions through data processing inequality (DPI). For each ARACNE-derived unweighted network, we further identified key regulators by examining the number of *N*-hop neighborhood nodes (NHNN) for each gene.

### Cell culture

Astrocytes were obtained from fetal C57BL/6 mice (embryonic day 18). We first used 75% alcohol to disinfect the mice for 5 min, with the mice then euthanized using cervical dislocation. Each brain was removed and placed in pre-cooled phosphate buffer solution (PBS), and the cerebral cortex was separated under an anatomic microscope and placed in DMEM/F12 medium. The cerebral cortex was then cut into pieces, after which 3 ml of 0.125% trypsin containing EDTA was added and digested at 37 °C for 8 min. Digestion was terminated with serum DMEM/F12. We then used 100-mesh cell filters to filter the tissue into a new centrifuge tube. Samples were centrifuged at 1000 rpm for 5 min, after which the supernatant was removed. Cells were suspended with serum DMEM/F12 (2% FBS + 1% PS + 1% star cell growth factor) and inoculated in 75 cm^2^ cell vials pretreated with PDL at a dose of 1 × 10^6^/ml. The cell suspension was placed in an incubator at 37 °C with 5% CO2. Culture medium was changed every 2–3 days. We identified astrocytes by microscope by their star shape, cobblestone mosaic arrangement, contact inhibition, and good light transmittance.

The day before infection, cells were plated in a 96-well plate at a cell density of 4 × 10^4^/well. Virus was added at a density of 1 × 10^8^ TU/ml and gently shaken in the “+” direction to evenly distribute the virus on the cell surfaces. The plate was then returned to the incubator for further incubation. After 24 h of virus infection, the cell culture medium was changed. We measured the infection efficiency of the virus by green fluorescence after 3 days.

### Mice

Sixteen male mice (c57-B6) were used in this study. The mice were group-housed (5–6 mice per cage) in an air conditioning-regulated environment (22–24 °C). Mice were kept in a 12-h light/dark cycle with ad libitum access to food and water. We started the experiment when the mice were 6 months old. All animal care and experimental protocols were approved by the Institutional Animal Care and Use Committee (IACUC) at the Kunming Institute of Zoology (approval number: SMKX2018021), Chinese Academy of Sciences.

### AAV injection

We injected AAV-CAG-PGLS-GFP vectors (serotype PHP.eb and titer = 4.17 × 10^12^ vg/ml) with hybrid CMV-chicken *β-*actin (CAG) promotor into AAV-PGLS mice (number = 8, age 6 months), and AAV-CAG-GFP vectors (serotype PHP.eb and titer = 8.2 × 10^12^ vg/ml) with hybrid CMV-chicken *β*-actin (CAG) promotor into Ctrl mice (number = 8, age 6 months), with each mouse injected with 4 × 10^11^ vg viral vectors. The number of GFP-positive cells showed no significant differences between AAV-PGLS and Ctrl mice (*p* = 0.1783), indicating similar virus expression efficiency in the two groups.

### Differential expression analysis of brains in AAV-PGLS and Ctrl mice

Brains from 2 AAV-PGLS mice (13 samples) and 2 Ctrl mice (11 samples) were used to extract RNA and RNA-seq as per the above method. The sequencing data were deposited in the Genome Sequence Archive database (http://gsa.big.ac.cn/) under accession ID CRA001751. The DESeq2 R package was used to identify DEGs [91], with a nominal significance threshold of *p* < 0.05. The *p* value was adjusted for multiple testing using Benjamini-Hochberg to estimate the FDR. We used g:Profiler (https://biit.cs.ut.ee/gprofiler/) to assess the enrichment of functional categories of DEGs.

### Western blot analysis

Brain tissue protein extracts were prepared with RIPA lysis buffer containing both protease and phosphatase inhibitors. Equal amounts of brain tissue lysates (80 μg) were loaded onto 12% sodium dodecyl sulfate (SDS)-polyacrylamide gel electrophoresis (PAGE) gels and transferred onto polyvinylidene difluoride (PVDF) membranes. After the membranes were blocked, they were incubated with monoclonal antibodies against PGLS (1:2000, Abcam), PSD95 (1:500, Abcam), and Caspase-3 (1:2000, Cell Signaling Technology) followed by incubation with HRP-Rb-anti-goat (1:2000, Beyotime) and *β*-actin (1:2000, Beyotime) followed by incubation with HRP-goat-anti-mouse (1:2000, Beyotime). Target proteins were detected by the ECL system (Millipore, Braunschweig, Germany) and visualized with the ChemiDoc XRS system (Bio-Rad, Hercules, CA, USA).

### Immunohistochemistry

After perfusion with PBS, the brains were removed and post-fixed in 4% paraformaldehyde at 4 °C overnight. Brain sections (40 μm) were cut with a vibrating blade microtome (Leica VT1000 S, Germany). Sections were washed for 1 h in PBS containing 5% bovine serum albumin (BSA) and 0.3% Triton X-100 and incubated with primary antibodies of anti-GFP (Invitrogen, 1:800), anti-Aβ40-42 (1:400; Millipore), anti-PGLS (1:200 NAVOUS), anti-GFAP (1:800 Cell Signaling), and anti-NeuN (1:800; Abcam) in PBS with 1% BSA and 0.3% Triton X-100 overnight at 4 °C, followed by incubation with corresponding secondary Cy3- and Cy2-conjugated antibodies (1:800; Jackson Lab) for 2 h at room temperature. Confocal z-stack images were acquired on a Nikon A1 confocal laser microscope system (Japan). Image J was used to count cell numbers, analyze fluorescence intensity of immunoreactive cells, and quantify GFAP morphology according to previous protocols [98]. Cell counts in the hippocampus, prefrontal lobe, temporal lobe, striatum, occipital lobe, and parietal lobe were performed in three randomly selected sections from each animal.

### Open field test

Experiments were performed between 16:00 pm and 18:00 pm. A Plexiglas box (27 × 27 × 20.3 cm, ENV 510) equipped with infrared beams and activity monitor (Med Associates, USA) was used in this test. To minimize background stress, mice were transported to the testing room 1 h prior to testing. After that, mice were placed at a fixed position in the chamber at the start of the assay and allowed to freely explore the chamber. The locomotor activity was monitored and recorded in the last 20-min period (previous 10 min for habituation).

### Morris water maze test

As previously described [99], mice were tested in a Morris water maze (120 cm diameter, 60 cm high), which was filled with water (22 °C) containing non-toxic titanium pigment to obscure the submerged platform (10 cm in diameter). Before training, all mice were adapted to the pool without a platform for 2 days (1 min/day). After that, mice were trained to find the hidden platform using distal extra maze cues. Mice were given four trials per day (60 s/trial with an inter-trial interval of 40 s). Each mouse was placed in the water with its head facing the pool wall, and the start point varied semi-randomly between trials. If the mouse did not find the submerged platform at the end of the trail, it was led to the platform by the experimenter, where it then remained for 15 s. Training was performed for five consecutive days, and latency to the platform was evaluated using the EthoVision 8.0 program (Noldus). The probe test (platform removed) was conducted for 1 min on day 6. The time spent in the four quadrants, number of platform crossings, and distance to platform were recorded.

### Rotarod test

Test mice were habituated to the testing room for 1 h. During the acceleration phase, mice were placed on the rotating rod (Panlab Harvard, Spain) with a 4-rpm constant speed for 10 s, with the apparatus then accelerated from 4 to 40 rpm in 5 min and the latency to fall recorded. Each mouse was tested three times with 40-min intervals.

### Metabolic test

Quantities of food intake were assessed by a metabolic cage (Panlab Harvard, Spain). Mice were individually placed in single cages and allowed free access to water and food during a 24-h period. Food intake was recorded and calculated by monitoring software (Panlab Harvard) each hour automatically. Feces in each metabolic cage were collected for weighing after the 24-h period, and body weight was measured manually.

### Statistical analyses

Data analysis was conducted using SPSS v19.0 (SPSS, Chicago, IL, USA) and GraphPad Prism v7.00 (GraphPad Software, La Jolla, CA, USA) in Windows. The *F* test was used to compare variances (*p* > 0.05), and normality was analyzed by the Shapiro-Wilk normality test (*p* > 0.05). All data acquisition and analysis were performed in a double-blind manner. Comparisons between two groups were conducted by unpaired *t* tests with Welch’s correction (normally distributed and variances differ), two-tailed unpaired-sample *t* tests (normally distributed and equal variances), or Mann-Whitney tests (non-normally distributed). Repeated-measure analysis of variance (ANOVA) was used for inter-group analysis. All data were expressed as means ± SEM, *p < 0.05, **p < 0.01, and ***p < 0.001.

## Supplementary information

**Additional file 1.** Supplementary Figures S1–S18.

**Additional file 2.** Supplementary Tables S1–S10.

**Additional file 3.** Review history.

## Data Availability

The raw RNA-seq data from the 590 samples of macaque brain were deposited into the Genome Sequence Archive database under accession ID CRA000336 (http://bigd.big.ac.cn/search?dbId=gsa&q=CRA000336) [100] and the NCBI Sequence Read Archive under bioproject ID PRJNA578504 [101] (https://www.ncbi.nlm.nih.gov/bioproject/PRJNA578504). The raw RNA-seq data of *PGLS* overexpression in mouse brains were deposited into Genome Sequence Archive database under accession ID CRA001751 (http://bigd.big.ac.cn/search?dbId=gsa&q=CRA001751) [102] and the NCBI Sequence Read Archive under bioproject ID PRJNA578197 [103] (https://www.ncbi.nlm.nih.gov/bioproject/PRJNA578197).
