# Author Correction: 547 transcriptomes from 44 brain areas reveal features of the aging brain in non-human primates

**DOI:** 10.1186/s13059-020-01962-z

**Published:** 2020-02-24

**Authors:** Ming-Li Li, Shi-Hao Wu, Jin-Jin Zhang, Hang-Yu Tian, Yong Shao, Zheng-Bo Wang, David M. Irwin, Jia-Li Li, Xin-Tian Hu, Dong-Dong Wu

**Affiliations:** 1 State Key Laboratory of Genetic Resources and Evolution, Kunming Institute of Zoology, Chinese Academy of Sciences, Kunming, 650223 Yunnan China; 2 Kunming College of Life Science, University of the Chinese Academy of Sciences, Kunming, 650223 Yunnan China; 3 Key Laboratory of Animal Models and Human Disease Mechanisms of Chinese Academy of Sciences & Yunnan Province, Kunming Institute of Zoology, Chinese Academy of Sciences, Kunming, 650223 Yunnan China; 4 Yunnan Key Laboratory of Primate Biomedicine Research, Institute of Primate Translational Medicine, Kunming University of Science and Technology, Kunming, 650500 Yunnan China; 5 Department of Laboratory Medicine and Pathobiology, University of Toronto, Toronto, ON Canada; 6 Center for Excellence in Brain Science and Intelligence Technology, Chinese Academy of Sciences, Shanghai, 200031 China; 7 National Research Facility for Phenotypic and Genetic Analysis of Model Animals, Kunming Institute of Zoology, Chinese Academy of Sciences, Kunming, 650223 Yunnan China; 8 Kunming Primate Research Center, Kunming Institute of Zoology, Chinese Academy of Sciences, Kunming, Yunnan China; 9 Center for Excellence in Animal Evolution and Genetics, Chinese Academy of Sciences, Kunming, 650223 Yunnan China

**Correction to: Genome Biol (2019) 20:258**

**https://doi.org/10.1186/s13059-019-1866-1**

Following publication of the original paper [1], the authors reported an error in the affiliation of Xin-Tian Hu, who is also affiliated with “Kunming Primate Research Center, Kunming Institute of Zoology, Chinese Academy of Sciences, Kunming, Yunnan, China”.
