# Supplementary material for: 547 transcriptomes from 44 brain areas reveal features of the aging brain in non-human primates
Source: Genome Biol. 2019 Nov 28;20:258. doi: 10.1186/s13059-019-1866-1 (PMC6883628; doi:10.1186/s13059-019-1866-1)
Supplement: Supplementary file 1 — Additional file 1. Supplementary Figures S1–S18. [file 13059_2019_1866_MOESM1_ESM.docx]

Supplementary Figures for

**547 Transcriptomes from 44 Brain Areas Reveal Features of the Aging Brain in Non-Human Primates**

Ming-Li Li^1, 3, #^, Shi-Hao Wu^2, 3, #^, Jin-Jin Zhang^1, 3^, Hang-Yu Tian^1, 3^, Yong-Shao^1, 3^ Zheng-Bo Wang^4^, David M Irwin^7^, Jia-Li Li^2^, Xin-Tian Hu^2, 5, *^, Dong-Dong Wu^1, 6, *^


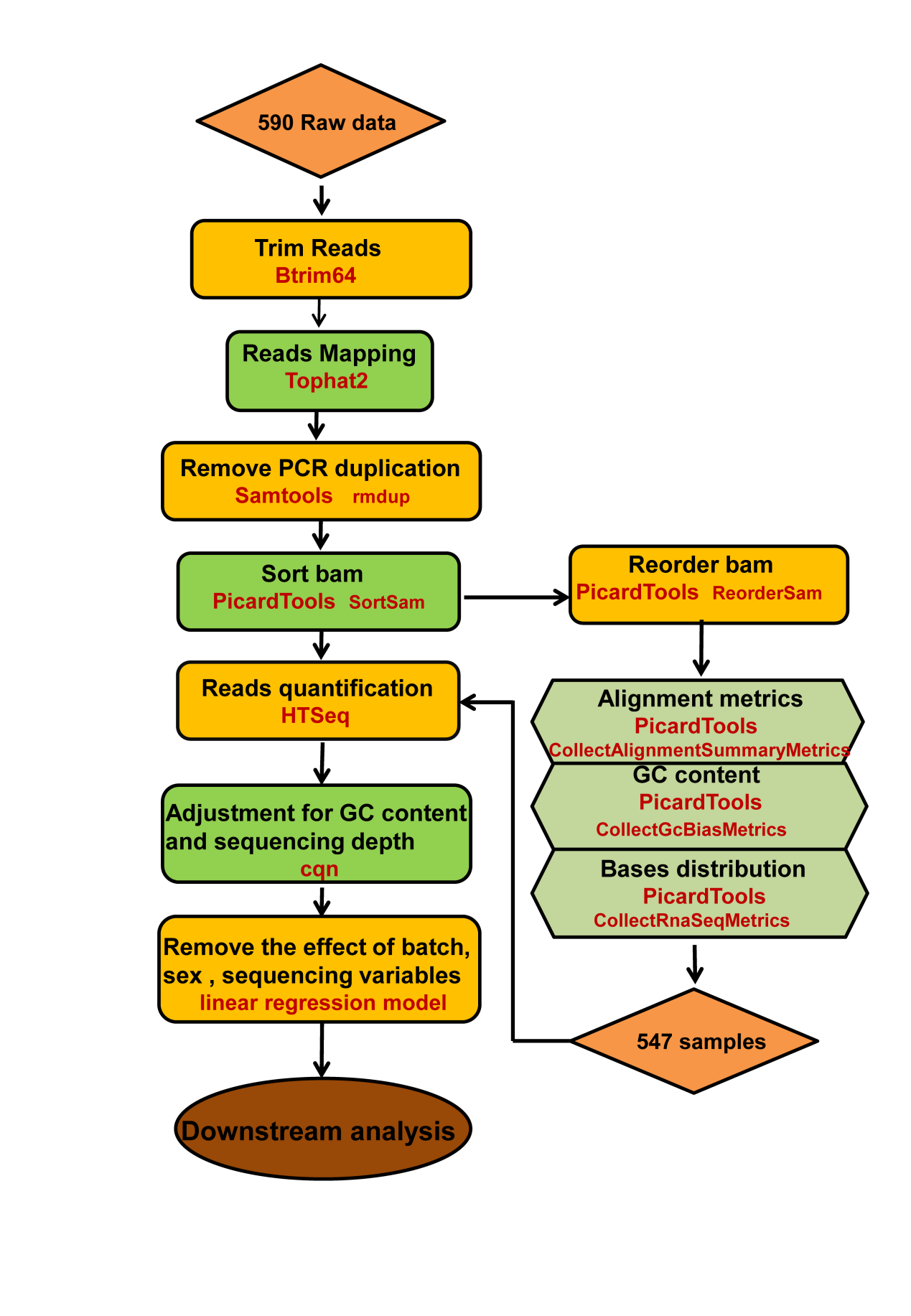


**Fig. S1.** Quality control workflow. See Methods for details.


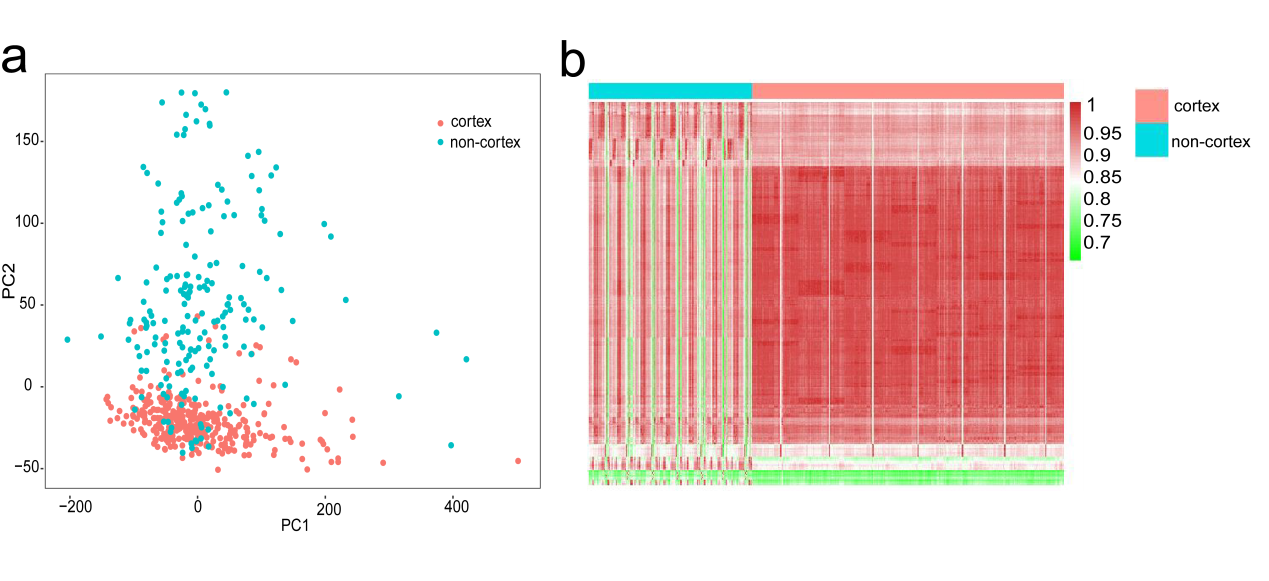


**Fig. S2. a**, Principal component analysis showing transcriptional similarity of cortex and non-cortex. **b**, Hierarchical clustering of RNA samples based on inter-array correlation showing distinct clusters of cortex and non-cortex.


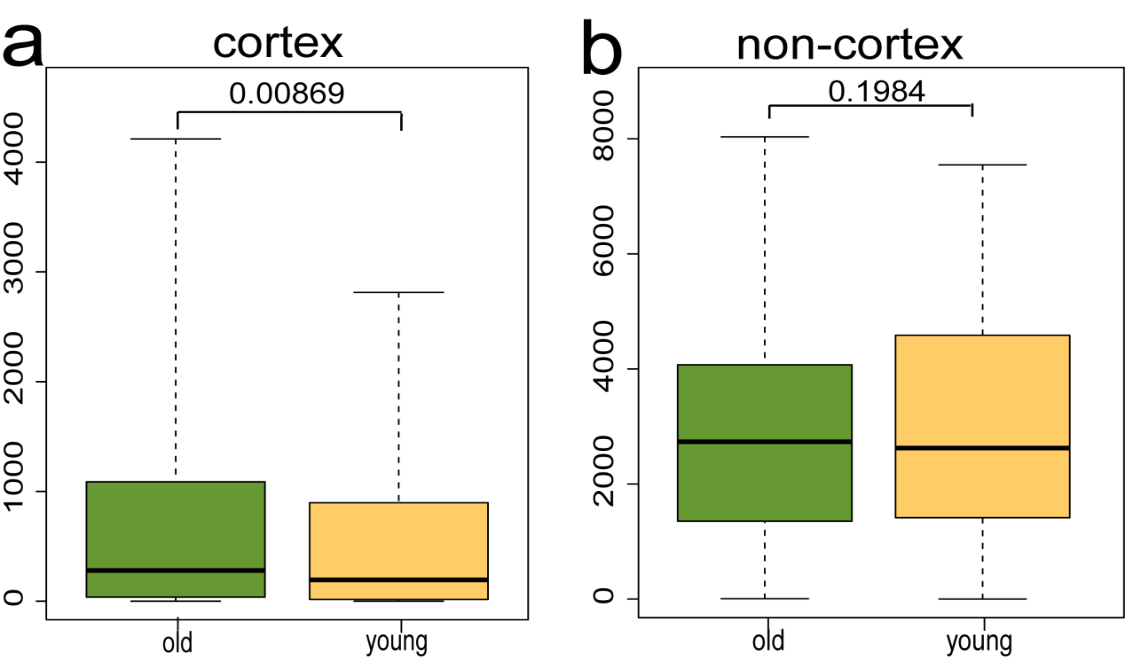


**Fig. S3.** Number of spatially differentially expressed genes in young and aged samples across cortex (a) and non-cortex (b).


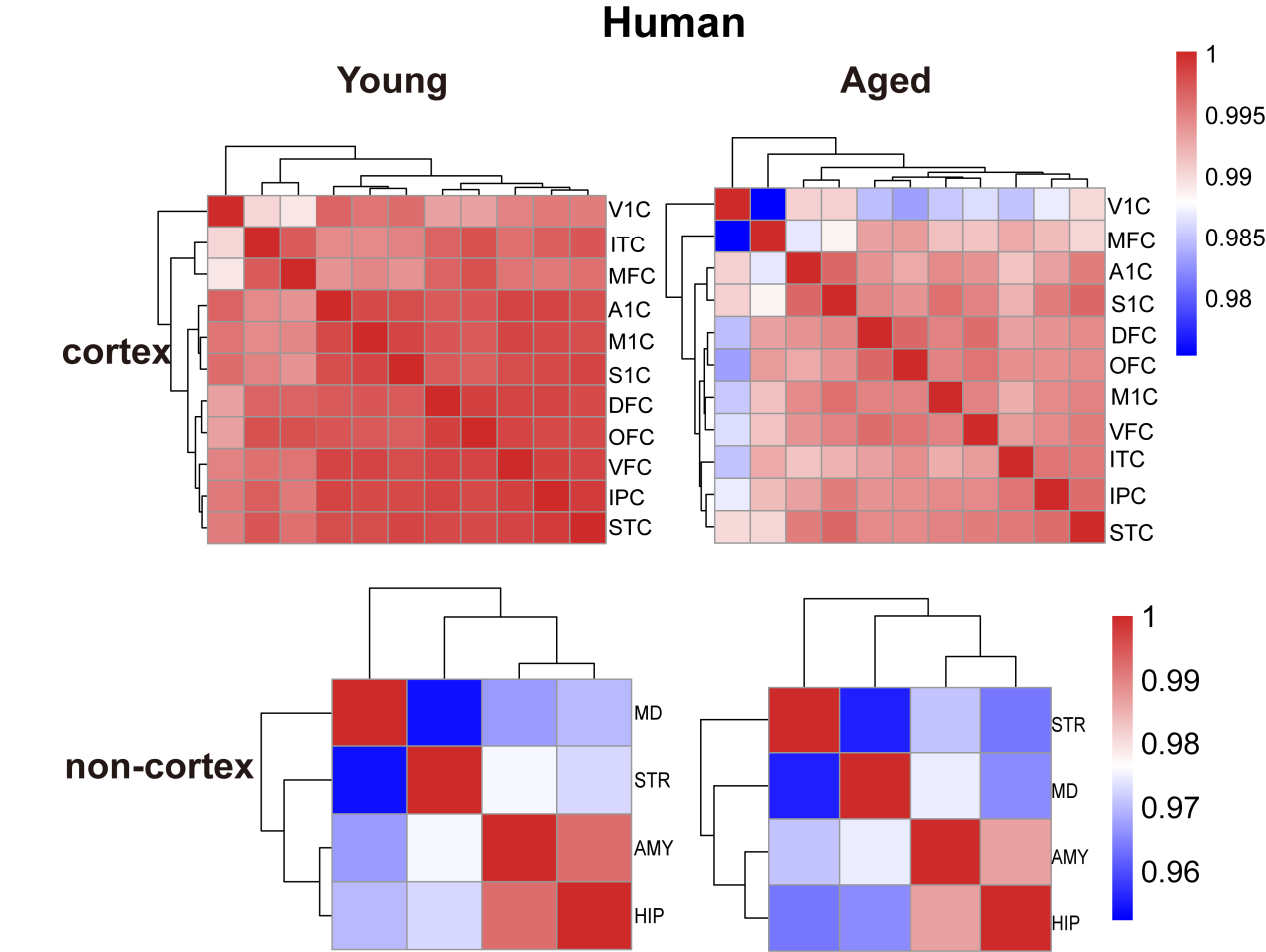


**Fig. S4.** Heatmap matrix of pairwise Pearson correlations between cortex regions (top) and between non-cortex areas (bottom) in humans.


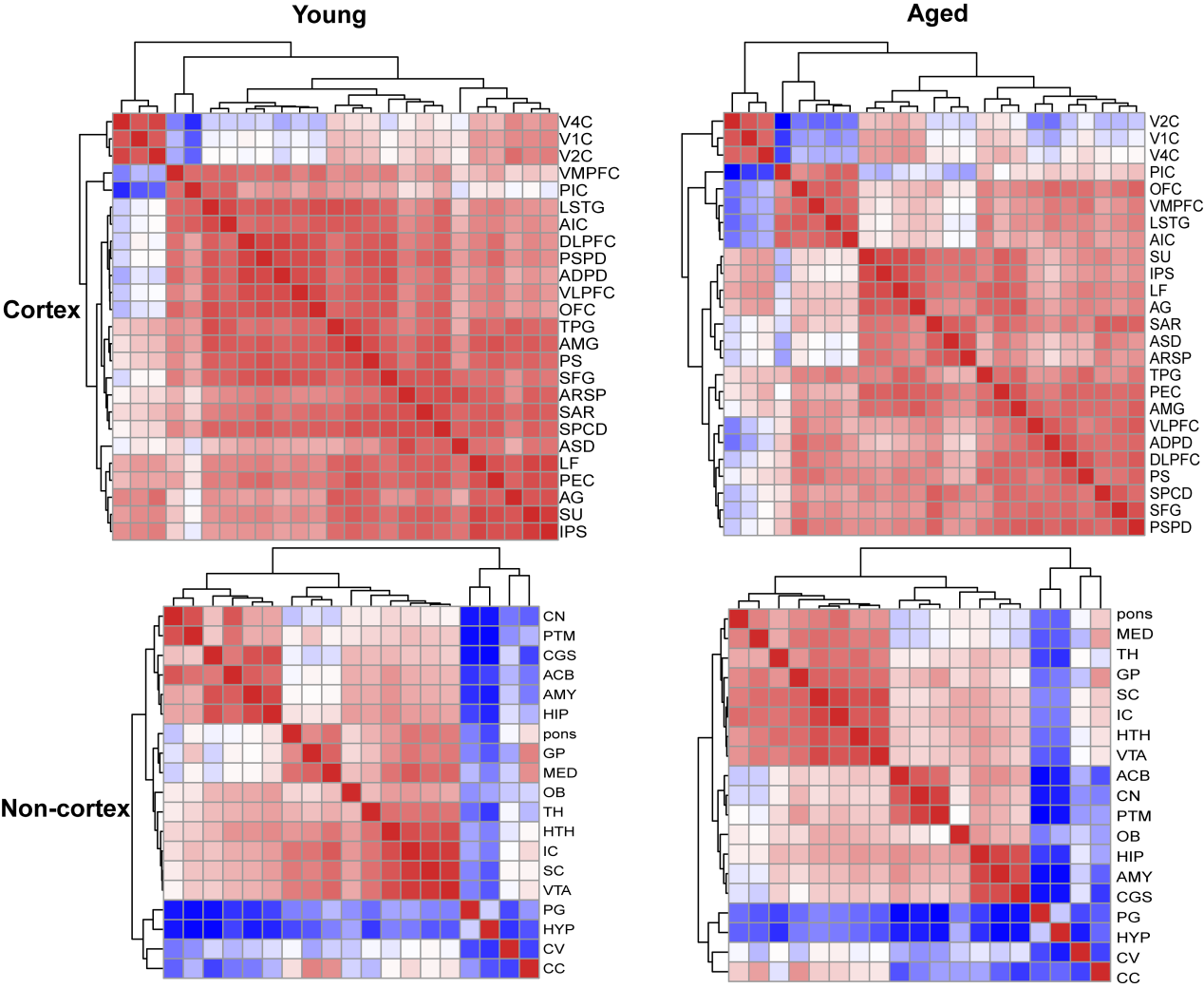


**Fig. S5.** Clustering of pairwise Pearson correlations between cortex regions (top) and between non-cortex areas (bottom) in young and aged macaques.


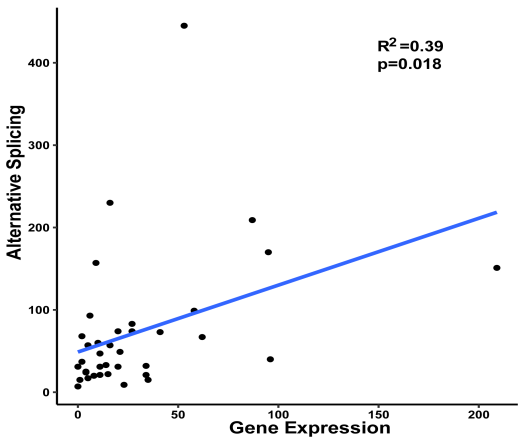

**Fig. S6.** Correlation plot of number of differentially expressed genes (DEGs) and genes with differential exon usage genes (DEUs) across brain regions (Pearson correlation test, a continuity correction is used for p value).


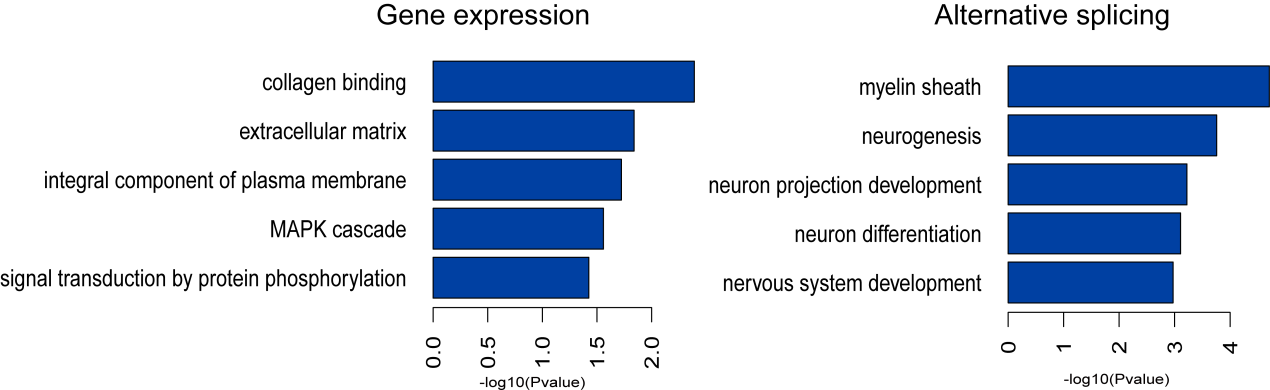


**Fig. S7.** Functional enrichment of genes exhibiting differential expression (left) and differential alternative splicing (right).


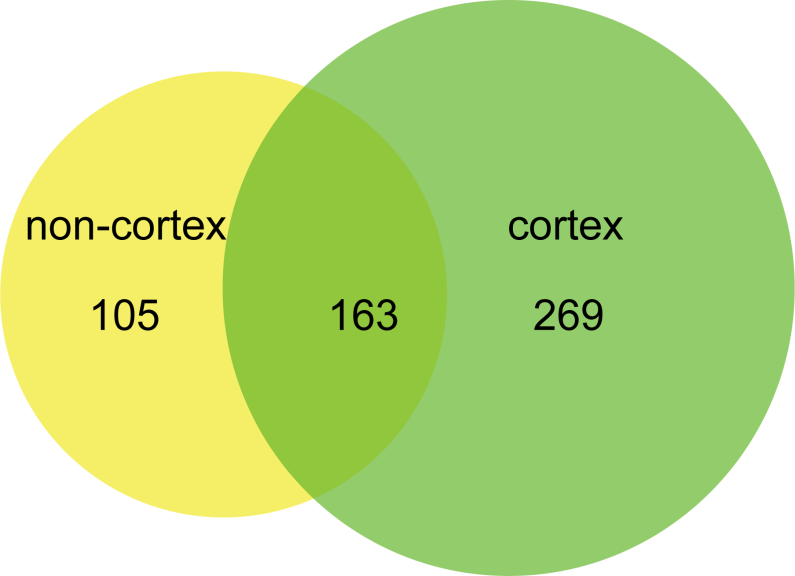


**Fig. S8.** Venn diagram depicting overlap between differentially expressed genes after aging in cortex and non-cortex.


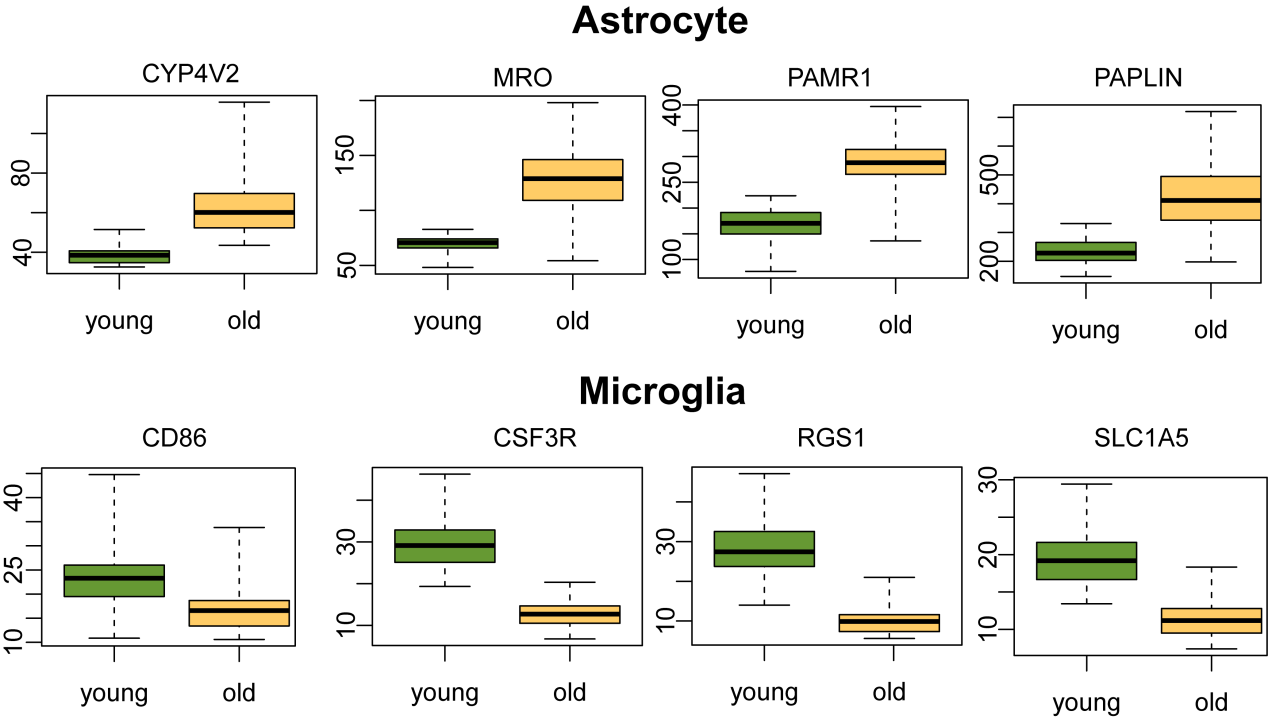


**Fig. S9.** Genes associated with astrocytes (upper) and microglia cells (below) that were differentially expressed in a large number of regions.


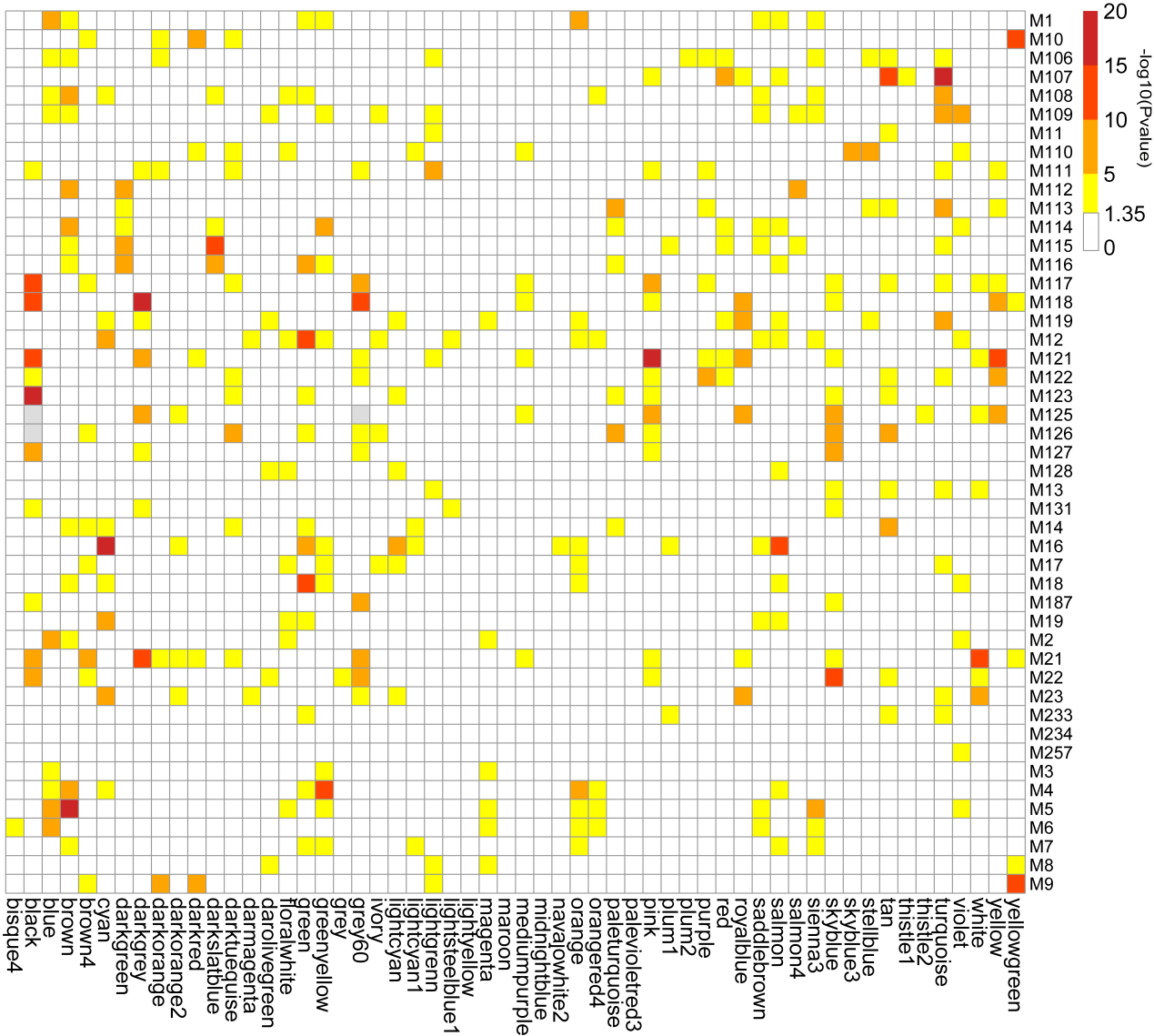


**Fig. S10.** Heatmap showing enrichment of modules in the current study and previously published dataset[[1](#_ENREF_1)].


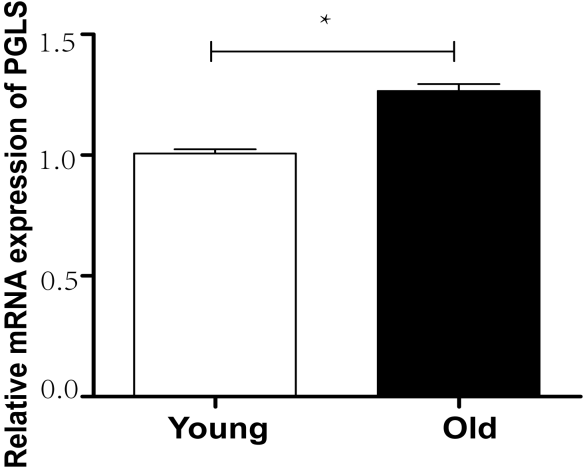


**Fig. S11.** Real-time quantitative polymerase chain reaction (qRT-PCR) analysis of PGLS mRNA expression in young and aged macaque brains (unpaired *t*-test t = 5.727, *p* = 0.029).


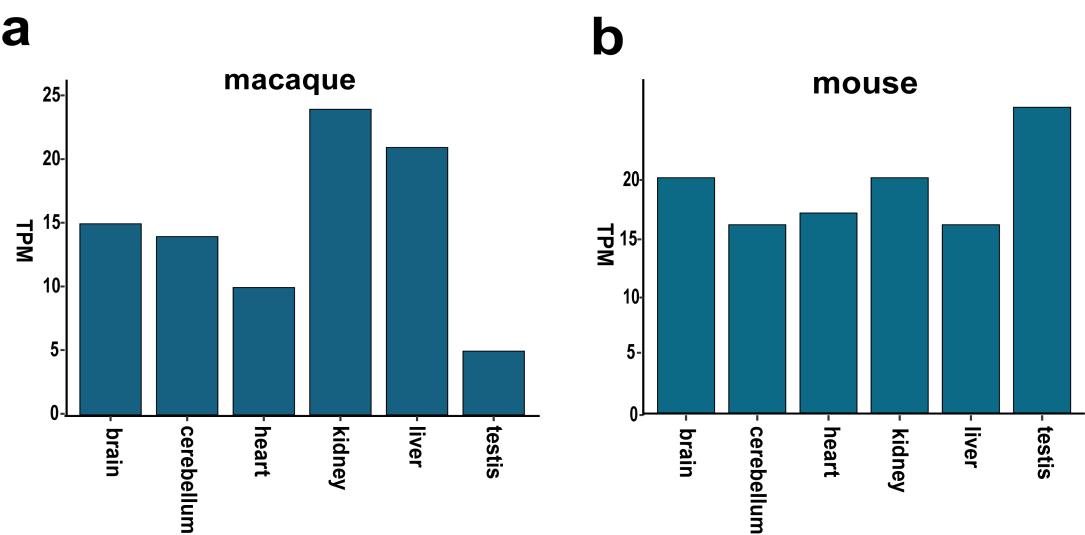


**Fig. S12.** Gene expression pattern of *PGLS* in six tissues in macaques (a) and mice (b).

**
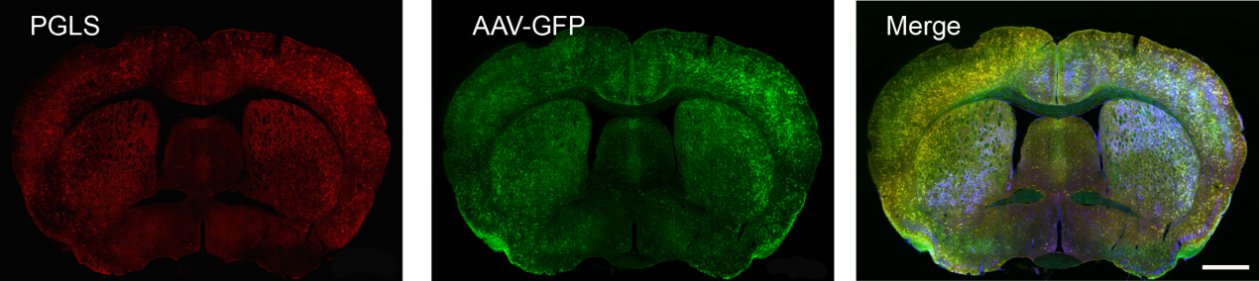
**

**Fig. S13.** Whole brain staining map in coronal position, bar = 1 mm, *PGLS*: With *PGLS* antibody staining, AAV-GFP: GFP tag carried by AAV used to overexpress *PGLS*.


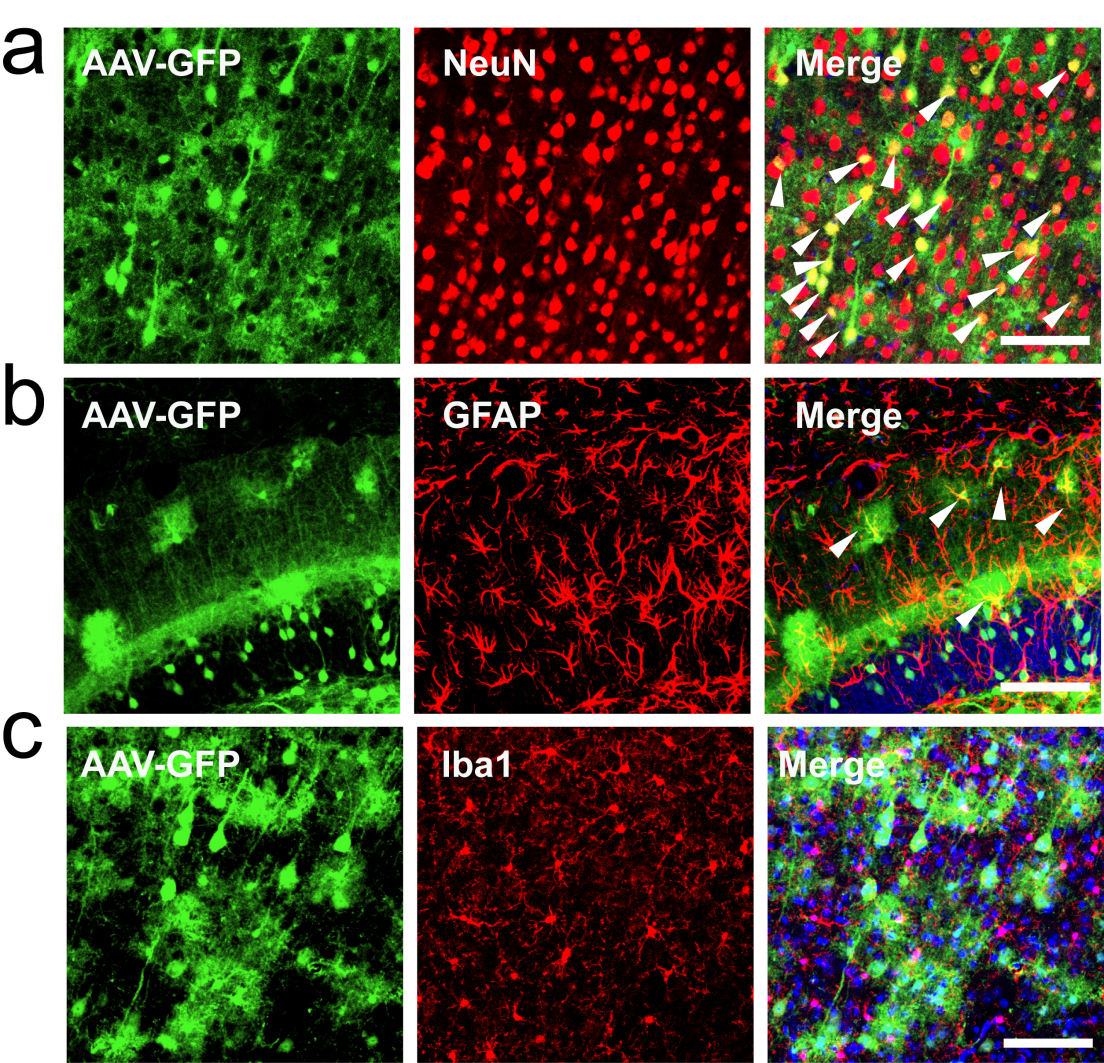


**Fig. S14. a.** Co-stained neurons (NeuN), **b.** astrocytes (GFAP), **c.** microglia cells (Iba1) with green fluorescence protein (GFP) tag co-expressed with *PGLS* in AAV virus, bar = 100 µm. White arrows indicate neurons and astrocytes transduced by AAV used to overexpress *PGLS*.


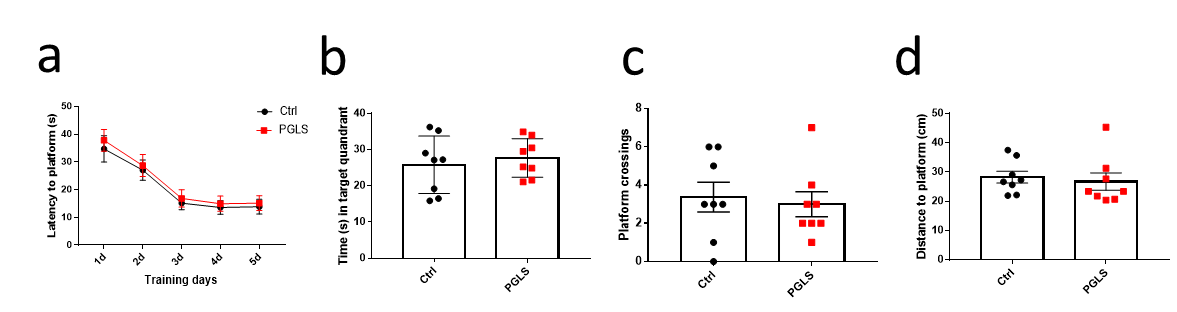


**Fig. S15.** Before virus injection, mouse groups showed no significant differences in the Morris water maze (MWM) task in training trials for (a) escape latency (repeated-measure ANOVA, F = 0.97, *** *p* = 0.983, ηp^2^ = 0.003) and in probe trials for (b) time in target quadrant (unpaired *t*-test t = 0.564, *p* = 0.5817), (c) platform crossings (Mann-Whitney test U = 27.5, *p* = 0.6772), and (d) distance to platform (Mann-Whitney test U = 23, *p* = 0.3823) (Ctrl: n = 8; AAV-PGLS: n = 8).

**Fig. S16.** Compare to Ctrl mice, AAV-PGLS mice displayed normal motor coordination and balance with the rotarod test. There were no significant differences between AAV-PGLS group (n = 8) and Ctrl group (n = 8) in the rotarod test (12 months; repeated-measure ANOVA, F = 1.070, *p* = 0.391, ηp^2^ = 0.113).


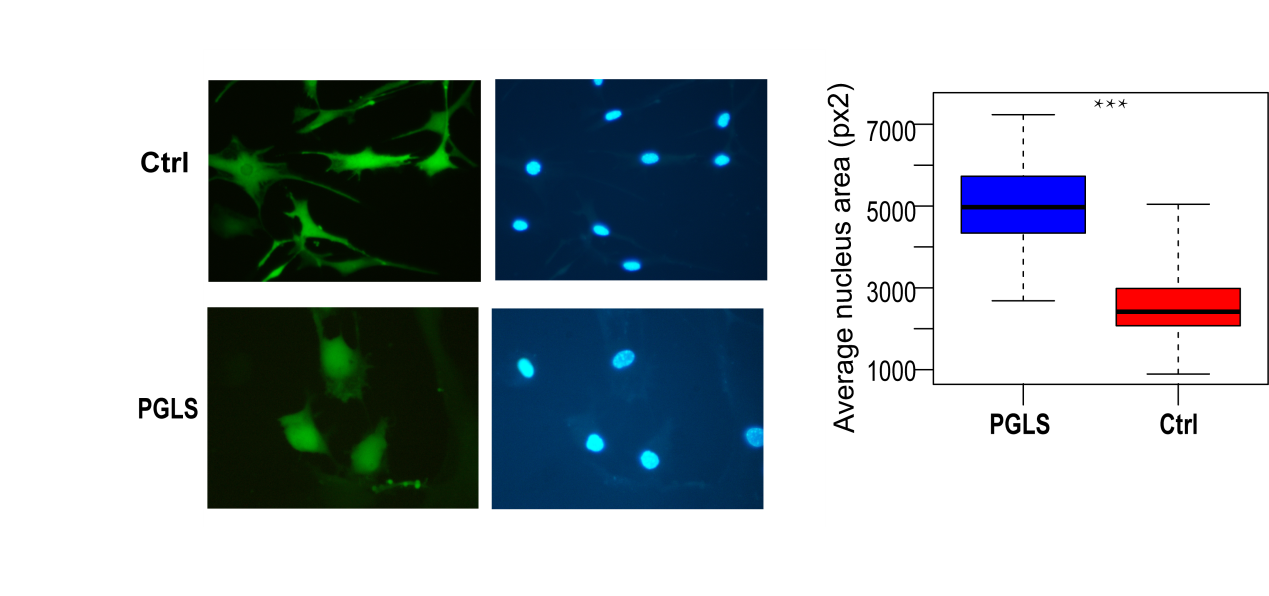


**Fig. S17** Nuclear size evaluation of up-regulated *PGLS* and Ctrl astrocyte cells after culture for 72 h. Size is expressed as squared pixels (px^2^). Values represent means ± SEM of n ≥ 6 independent experiments. ****p* < 0.001 (unpaired *t*-test).


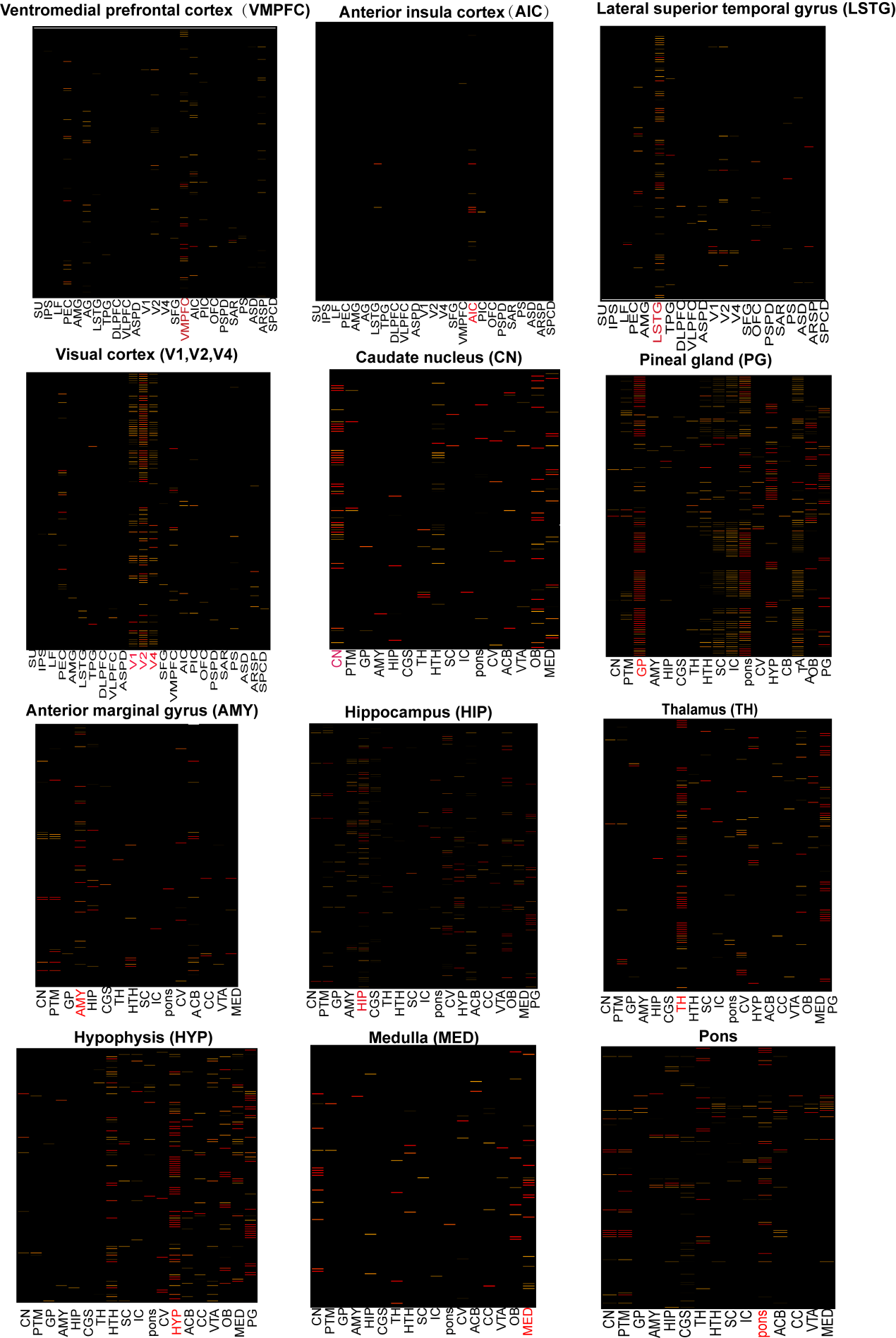


**Fig. S18.** Heatmap showing expression of marker-gene list of specific brain regions[[2](#_ENREF_2)].

**References**

1. Mostafavi S, Gaiteri C, Sullivan SE, White CC, Tasaki S, Xu J, Taga M, Klein HU, Patrick E, Komashko V, et al: **A molecular network of the aging human brain provides insights into the pathology and cognitive decline of Alzheimer's disease.** *Nat Neurosci* 2018, **21:**811-819.

2. Hawrylycz MJ, Lein ES, Guillozet-Bongaarts AL, Shen EH, Ng L, Miller JA, van de Lagemaat LN, Smith KA, Ebbert A, Riley ZL, et al: **An anatomically comprehensive atlas of the adult human brain transcriptome.** *Nature* 2012, **489:**391-399.
