# Supplementary material for: 547 transcriptomes from 44 brain areas reveal features of the aging brain in non-human primates
Source: Genome Biol. 2019 Nov 28;20:258. doi: 10.1186/s13059-019-1866-1 (PMC6883628; doi:10.1186/s13059-019-1866-1)
Supplement: Supplementary file 3 — Additional file 3. Review history. [file 13059_2019_1866_MOESM3_ESM.docx]

Review History

**First round of review**

**Reviewer 1**

**Are you able to assess all statistics in the manuscript, including the appropriateness of statistical tests used?**

Yes, and I have assessed the statistics in my report.

**Comments to author:**

In this work, authors studied gene expression profiles in various brain regions of young and old monkeys. With WGCNA analysis, author identified a critical hub gene -PGLS. Authors showed that PGLS is an aging-related gene and may play a role in brain aging process rodents. This work started from an unique angle to study brain aging and shed new insights on molecular mechanisms of aging. I have following comments, 

Major comments:
1. Authors have unique resources of access to young and old monkey at the same time. For cognitive function, young (6-7 y old) and old (16-24 y old) monkeys may have some difference which could be explained by the gene expression profiles. In particular, some learning and memory tasks are associated with some specific brain regions. Given the details gene expression database, authors should provide more in-depth analysis or discussion in this regard. 
2. The identification of PGLS gene is particularly interesting. But several critical issues have to be addressed. First, what is the endogenous expression pattern of PGLS in primate or in rodent? If it is not a primate specific gene, this question could be address in rodent first. 
3. The most important experiment seems to me, is to identify whether PGLS is expressed in neuron or glial cells? Author used elegant AAV-php-eb virus to overexpress PGLS in mouse neuron, driven by the synapsin promoter. This result suggests that PGLS in neurons may cause aging. However, author went on to show that overexpression of PGLS also causes activation of astroglia cells. So whether PGLS works in neuron or glia or both types of cells needs to be clearly addressed. PGLS could be a widely expressed gene, but authors should present this important piece of data. 

Minor comments:
1. With AAV-php-eb virus, authors need to show the expression pattern in the brain in more details, for example, is it in cortical or sub-cortical distribution?
2. With glia activation author observed, it is worthy to examine whether microglia is also activated in the PGLS-overexressing brain, since microglia activation is also a critical sign for neural inflammation.

**Reviewer 2**

**Are you able to assess all statistics in the manuscript, including the appropriateness of statistical tests used?**

Yes, and I have assessed the statistics in my report.

**Comments to author:**

The manuscript by Li et al. reports the transcriptomes of 44 brain regions in young and old macaques in order to determine the transcriptional changes associated with aging in the primate. In the cortical areas studied, the authors observe a decrease in the transcriptional profile correlation between brain areas as well as between hemispheres associated with aging. Analysis of an independent dataset in human confirms this result. Non-cortex regions do not seem to be affected by aging. Both levels of gene expression and alternative splicing are found altered in aging; these changes are widespread in the brain but their degree is area-specific. Co-expression network analysis defined 56 modules among which the connectivity of 9 is increased in aging. Functional analysis reveals that they include various annotation groups that confirm the idea of a multifactorial aspect of aging. Fine analysis of the most affected module shows deep reorganization of the network with nearly half of the hub genes being age-specific. Finally, among the hub genes appearing with aging, the authors decided to focus on PGLS, part of the pentose phosphate pathway, and to validate its role in aging in the mouse. Overexpression of PGLS in the mouse brain via viral vector induces histological, behavioral, and cognitive signs of aging. The authors propose PGLS as a novel biomarker of aging.

Overall, this is an interesting study that builds on a wide array of experimental and modeling methods to look for a transcriptional signature of aging in the primate brain and conclude by successfully validating one of their candidate genes with various cellular and behavioral assays in the mouse. It could also be a great data resource for scientists in the field of aging. 
Unfortunately, in its current state, the lack of clarity (1) in some critical methods and (2) in the way the results are reported/ presented makes it difficult to evaluate and, at time, diminish its interest.
Major comments:
1.     Methods:
-Brain dissection: Brain region samples are the primary material of this study. However, this procedure is not described at all in the methods section. 
Which reference atlas has been used? 
How the anatomical changes (growth) between the 2 age groups have been corrected? 
How was the quality of the dissection validated? RNAseq data makes it easy to verify the presence of molecular markers specific to a given region. 
What were the delays between time of death, brain extraction, completion of the sampling…?

-RNAseq analysis:
The authors claim to have normalized RNAseq datasets. However, there is no description of the procedure either in the text or in the methods. The data are only mentioned as "fragments" or "reads".
Normalization is essential for data processing and comparison of gene expression both between brain regions and individuals. The read counts have to be normalized for sequencing depth and for potential batch effect between individuals.

-Statistics: 
MWU tests are performed to compare DEG as well as the correlations between area and hemispheres but there is no mention of adjusted p-values for multiple testing in the methods.  Adjustment is only described for the alternative splicing (DEU) analysis.

2.     Results:
-Overall, the manuscript language is lacking precision; to the point that, at time, there seems to be overstated claims.
For example, the claim of alteration of the metabolic rate of the PGLS transduced mice where one would expect to find a difference in energy expanded over a certain period of time but only get a measure of food intake and dejection weights. 
The fact that PGLS overexpression induces synapses loss and increased apoptosis does not necessarily mean that synapses loss is caused by a higher rate of apoptosis.
The authors should clearly mention in the abstract and the discussion that PGLS validation was is done in the mouse.

-Figure1: The diagram nicely summarizes the strategy used in the manuscript. Maybe a separate diagram reporting the validation of PGLS in the mouse would make it even clearer.
There is no mention in the legend to the regions colored in green, blue, and red; what are they referring to?

-Figure2: The heatmap matrices would be more informative and the age-dependent alterations more visible if the matrices reporting the inter areas and inter hemispheres correlations were built with the brain regions ordered the way in the young and old groups.

-Figure3: (a) Is there some correlation between DEG and AS across brain regions?
(b, c, d) These 3 heatmaps are flattened by a few extreme p-values and consequently not very informative. For b and c, numbers or relative numbers of shared DEG between pair of regions would make the heatmaps more legible. Regarding (d), why not a correlation plot?
(f) A few examples of genes associated with each cellular type and affected in a large number of regions would be more informative.  

-Figure4: (f) The edges and the cyan dot are not visible. 

-Figure5: (i) Open field: the representative traces seem to show the inverse of the histograms.
(j) Food intake: on this panel PGLS mice are in black while they are in red in all other panels.

Minor comments:
1.     Overall, the manuscript is still full of typos and misused words (for example several occurrences of "alternations" for "alterations" lines 57, 82…). It would greatly benefit from a native speaker editing

2.     "from 3 young and 4 aged rhesus macaques" (line 80) instead of 4 young and 4 old macaques.

3.     While the analysis of a published human dataset is interesting (lines127-133), a few descriptive sentences regarding the age range of the subjects and the brain areas sampled would help.

4.     Mouse RNAseq experimental details and analysis are missing in the methods section.

5.     A sentence about macaque life stages and life expectancy would help the reader to put the 2 experimental groups in context.

6.     The figures resolution is too low and makes it difficult to evaluate.

7.     What was the rational between choosing 6-month-old mice (at the beginning) instead of standard 2 months mice? One would expect an even greater contrast with younger adults.

**Authors Response**

**Point-by-point responses to the reviewers’ comments:**

Comment

*Response*

Reviewers’ Comments, Author Responses, and Manuscript Changes

Reviewer #1:

In this work, authors studied gene expression profiles in various brain regions of young and old monkeys. With WGCNA analysis, author identified a critical hub gene -PGLS. Authors showed that PGLS is an aging-related gene and may play a role in brain aging process rodents. This work started from an unique angle to study brain aging and shed new insights on molecular mechanisms of aging.

*Reply:*

*Thank you for your time reviewing our manuscript. We sincerely appreciate your valuable comments, which have helped us to improve our manuscript. Please see our revisions in the revised manuscript, and our responses to your comments below.*

I have following comments,

Major comments:

1. Authors have unique resources of access to young and old monkey at the same time. For cognitive function, young (6-7 y old) and old (16-24 y old) monkeys may have some difference which could be explained by the gene expression profiles. In particular, some learning and memory tasks are associated with some specific brain regions. Given the details gene expression database, authors should provide more in-depth analysis or discussion in this regard.

*Reply:*

*Thank you very much for your advice. We agree that gene expression differences between young and aged monkeys could explain their different cognitive function to some extent, and that gene expression patterns for each brain region provide information on the special function of this region. According to your comments, we have added in-depth discussion on these issues in the revised manuscript, as follows:*

*“Based on comparison of gene expression profiles between young and old macaques, we found a decreased tendency of expression correlation among multiple brain areas after aging. Furthermore, combined with previous conclusions that expression correlations between major brain areas increase during human early brain development[28], we hypothesized that connectivity within the brain exhibits a “mountain-like” pattern across a lifespan: i.e., connectivity first increases during early brain development and then decreases with aging. In addition, the changes of expression profile after aging would reveal to some extent declines of brain functions, e.g. cognitive function. For example, it is well known that multiple brain areas must work together to accomplish complex cognitive functions[77]. Our results indicated that the connectivity between brain regions decreased after aging, which was consistent with loss of cognitive function in the process of brain aging.”*

*“Among the 44 brain regions analyzed, the putamen was highlighted as showing dramatic transcriptional changes during aging. However, few studies have focused on this region previously. The putamen is located at the base of the forebrain and together with the caudate nucleus forms the dorsal striatum[**77**]. Furthermore, it is reported to play an important role in cognitive functions, including learning, decision making, and motor behaviors[**78-80**], and is implicated in various neurological diseases, such as PD, AD, and HD[**32**,* *77**]. Our results suggest that the putamen should receive greater attention in future research, diagnosis, and treatment of brain aging. In addition to the putamen, other brain regions account for special functions and tasks, like learning, memory, and language. The large-scale transcriptome data obtained from the multiple brain regions in this study should provide insight into the functional changes that occur after aging for different regions based on changes in expression profiles.”*

2. The identification of PGLS gene is particularly interesting. But several critical issues have to be addressed. First, what is the endogenous expression pattern of PGLS in primate or in rodent? If it is not a primate specific gene, this question could be address in rodent first.

*Reply:*

*Thank you very much for your suggestion. PGLS is a conserved gene among mammals [**1**] and is expressed endogenously in both macaques and mice (Figure S12)[**2**]. We have added this description in the revised paper to make it easier to follow.*

*We identified PGLS as a candidate gene for brain aging by transcriptional analysis in macaques. However, as mice can be tested over a shorter period and with easier operation, we first selected mice as experimental animals to test the role of PGLS in brain aging. Future experiments on PGLS function in primates are necessary, which has been added to the revised paper, as follows:*

*“We propose that PGLS should be regarded as a novel biomarker of brain aging in future research. We confirmed the function of PGLS in mice; however, future experiments on PGLS in primates are necessary to further confirm the functions of this gene”*

**Figure S12**: Gene expression patterns of *PGLS* in six tissues in macaques (a) and mice (b).

3. With AAV-php-eb virus, authors need to show the expression pattern in the brain in more details, for example, is it in cortical or sub-cortical distribution?

*Reply:*

*Thank you very much for pointing out this issue. We have performed further experiments and provided this information in the revised paper. We have added Figure S13 in the Supplementary Material to present the expression pattern of the AAV-php-eb virus in the whole brain of mice. Results showed that the AAV-php-eb virus was transduced in both cortical and sub-cortical areas.*

**Figure S13** Whole brain staining map in coronal position, bar = 1 mm, *PGLS*: With *PGLS* antibody staining, AAV-GFP: GFP tag carried by AAV used to overexpress *PGLS*.

3. The most important experiment seems to me, is to identify whether PGLS is expressed in neuron or glial cells? Author used elegant AAV-php-eb virus to overexpress PGLS in mouse neuron, driven by the synapsin promoter. This result suggests that PGLS in neurons may cause aging. However, author went on to show that overexpression of PGLS also causes activation of astroglia cells. So whether PGLS works in neuron or glia or both types of cells needs to be clearly addressed. PGLS could be a widely expressed gene, but authors should present this important piece of data.

*Reply:*

*Thank you very much for your comment. As said, PGLS is a widely endogenous gene expressed across multiple cell types in mouse brains[**3**] (Fig. unpublished 1). We apologize for the error in the manuscript. We used hybrid* *CMV-chicken β-actin (CAG) promotor rather than synapsin promoter. The CAG promoter is broad spectrum and can induce AAV infection in multiple cell types[**4**]. According to the comment, we co-stained neurons (NeuN) and astrocytes (GFAP) with the GFP tag co-expressed with PGLS in the AAV virus. Results showed that AAV transduced both neurons and astrocyte cells (Figure S14 a~b), suggesting that PGLS probably worked in both neurons and astrocytes to induce brain aging.*

**Fig. unpublished 1** Endogenously expressed pattern of *PGLS* in astrocyte, neuron, microglia, and endothelia cells in mouse brain.

**Figure S14. a.** Co-stained neurons (NeuN), b. astrocytes (GFAP), c. microglia cells (Iba1) with green fluorescence protein (GFP) tag co-expressed with *PGLS* in AAV virus, bar = 100 µm. White arrows indicate neurons and astrocytes transduced by AAV used to overexpress *PGLS*.

4 With glia activation author observed, it is worthy to examine whether microglia is also activated in the PGLS-overexpressing brain, since microglia activation is also a critical sign for neural inflammation.

*Reply:*

*We thank the reviewer for pointing out this issue. According to your suggestion, we performed further experiments and co-stained microglia cells (Iba1) with the GFP tag co-expressed with PGLS in AAV virus (Figure S14c). We did not find microglia cells were transduced by AAV in this study. This result is consistent with previous research, whereby* *high cyclic GMP-AMP synthase (cGas) was identified in microglial cells, which can inhibit AAV transduction of microglial cells by the cGAS-STING signaling pathway[5]. Furthermore, we found no significant changes in microglia cells between the PGLS and Ctrl groups (Fig. unpublished 2), and morphological analysis also demonstrated that, compared to the Ctrl group, PGLS microglia cells were not* *morphologically altered by inflammation (Fig. unpublished 3)[6].*

**Fig. unpublished 2** Co-stained microglia cells (Iba1) with green fluorescence protein (GFP) tag co-expressed with *PGLS* in AAV virus, bar = 100 μm.

**Fig. unpublished 3.** Photomicrograph of fluorescent IHC and cropped microglia cell (Iba1) with skeletonized image. Compared to Ctrl group (n = 10 cells), AAV-PGLS group (n = 10 cells) showed no significant differences in endpoints/cell (*p* = 0.73) or process length/cell (*p* = 0.123), which are important morphological indicators of microglial inflammation [6]. Data are means ± SEM.

Reviewer #2: The manuscript by Li et al. reports the transcriptomes of 44 brain regions in young and old macaques in order to determine the transcriptional changes associated with aging in the primate. In the cortical areas studied, the authors observe a decrease in the transcriptional profile correlation between brain areas as well as between hemispheres associated with aging. Analysis of an independent dataset in human confirms this result. Non-cortex regions do not seem to be affected by aging. Both levels of gene expression and alternative splicing are found altered in aging; these changes are widespread in the brain but their degree is area-specific. Co-expression network analysis defined 56 modules among which the connectivity of 9 is increased in aging. Functional analysis reveals that they include various annotation groups that confirm the idea of a multifactorial aspect of aging. Fine analysis of the most affected module shows deep reorganization of the network with nearly half of the hub genes being age-specific. Finally, among the hub genes appearing with aging, the authors decided to focus on PGLS, part of the pentose phosphate pathway, and to validate its role in aging in the mouse. Overexpression of PGLS in the mouse brain via viral vector induces histological, behavioral, and cognitive signs of aging. The authors propose PGLS as a novel biomarker of aging.

Overall, this is an interesting study that builds on a wide array of experimental and modeling methods to look for a transcriptional signature of aging in the primate brain and conclude by successfully validating one of their candidate genes with various cellular and behavioral assays in the mouse. It could also be a great data resource for scientists in the field of aging.
Unfortunately, in its current state, the lack of clarity (1) in some critical methods and (2) in the way the results are reported/ presented makes it difficult to evaluate and, at time, diminish its interest.

*Reply:*

*Thank you for your time reviewing our manuscript. We sincerely appreciate your valuable comments, which have helped us to improve our manuscript. Please see our revisions in the revised manuscript, and our responses to your comments below.*

Major comments:
1. Methods:
-Brain dissection: Brain region samples are the primary material of this study. However, this procedure is not described at all in the methods section.
Which reference atlas has been used?

*Reply:*

*Thank you for pointing out this issue. We have provided more detailed information regarding this in the revised manuscript.*

*“According to a widely used macaque brain atlas (*[*http://www.brainmaps.org*](http://www.brainmaps.org)*), tissues spanning 44 anatomically distinct regions were selected and collected by a skilled technician* *with over a decade of experience, and he is also an operator of brain dissection in another studies[**81**,* *82**]. We applied a list of previously published gene markers specific to human brain regions* *to validate the accuracy of the brain dissection[**83**] (Additional file 1: Figure S18). Brain dissection of the seven macaques was performed from fresh specimens by the same person to ensure consistency in sampling between specimens. Surgical instruments were sterilized in advance and surgical scissors and tweezers were only used one time for each sample to avoid cross-contamination. Only the central portion of each brain region was sampled. Each sample consisted of 100 mg of dissected tissue. All collected samples were washed with* *RNAlater solution (AM7021, Ambion, USA) and placed in freezing tubes for storage at liquid nitrogen temperature.”*

How the anatomical changes (growth) between the 2 age groups have been corrected?

*Reply:*

*Thank you for pointing it out. The anatomical structure of macaque brain is largely steady* *at one year old based on MRI analysis[7] Thus, we believe that the anatomical structure of each region should be almost the same between the young and aged monkeys we analyzed, although it is very hard to exclude the confounding factor of anatomical changes completely.*

How was the quality of the dissection validated? RNAseq data makes it easy to verify the presence of molecular markers specific to a given region.

*Reply:*

*We have added a table in Additional file 2 to show the RNA integrity number (RIN) of the 590 samples (Table S1). Quality control of RNA was based on RIN, with one sample not fulfilling the cutoff of RIN ≤5, which is the criterion used in previous study[8].*

*According to your comment, we applied a list of previously published gene markers for human brain regions [9], which provides the most comprehensive data reporting gene markers of specific brain regions to our knowledge. The heatmap shows high expression of these marker genes in corresponding macaque brain regions (Figure S18), thereby validating the accuracy of the brain samples we collected. We have provided the analyses in the revised Supplementary Materials.*

Figure S18: Heatmap of gene-marker expression patterns across 12 major human brain regions in corresponding macaque brain regions.

What were the delays between time of death, brain extraction, completion of the sampling…?

*Reply:*

*After each macaque was euthanized, we collected the brain samples as soon as possible. Thus, there are almost no delay between time of death, brain extraction, and completion of sampling, although we cannot exclude this confounding factor absolutely. In addition, nearly all samples exhibited a high level of RNA quality based on RIN values, except one sample, which was excluded.*

-RNAseq analysis:
The authors claim to have normalized RNAseq datasets. However, there is no description of the procedure either in the text or in the methods. The data are only mentioned as "fragments" or "reads". Normalization is essential for data processing and comparison of gene expression both between brain regions and individuals. The read counts have to be normalized for sequencing depth and for potential batch effect between individuals.

*Reply:*

*Thank you. We have described the normalization of our RNA-seq dataset more clearly in the revised paper. We have also provided a flow chart in the Supplementary Material to clarify our analysis pipeline.*

Figure S1 Quality control workflow. See methods for details.

*We also provide the adjustment process of read counts as follows:*

*“Read alignment and quality control*

*We acquired 590 transcriptomes across 44 brain regions from four young and three aged macaques. First, quality control (QC) of RNA was performed based on the RIN (see Additional file 2, Table S1), with one sample excluded after failing our cutoff of RIN ≤ 5. We next use Btrim64 to trim reads to obtain high-quality reads[**84**]. The paired-end reads were mapped to the macaque reference genome using Tophat2[**85**]. The* rmdup *command in Samtools was used to remove PCR duplication of bam files[**86**], with the SortSam command in PicardTools (*[*http://broadinstitute.github.io/picard/*](http://broadinstitute.github.io/picard/)*) then used to sort bam files.*

*After read alignment, QC analysis was performed using PicardTools v1.100 (commands ReorderSam, CollectAlignmnetSummaryMetrics, CollectRnaSeqMetrics, CollectGcBiasMetrics). Sequencing metrics were used to remove samples with poor sequence quality based on the following sequencing metrics: %Total Reads, %High-quality Aligned Reads, %mRNA Bases, %Intergenic Bases, Median 5´ to 3´ Bias, GC Dropout, and AT dropout (**Additional file 2, Table S1). To detect outliers, a quality z-score was calculated for each metric, and samples with low quality (Z > 2 for %Intergenic Bases, GC Dropout, or AT Dropout and Z < -2 for %Total Reads, %High-quality Aligned Reads, %mRNA Bases, or Median 5’ to 3’ Bias) in this matrix were identified as outlier values, and any sample with greater than one outlier value was removed due to sequencing quality concerns. QC analysis was performed for the 590 initial samples, with 43 samples (7%) thus removed. The remaining 547 samples were used for downstream analysis (Additional file 1, Table S1).*

*Quantification and adjustment of gene expression*

*Gene expression levels were quantified for samples passing QC using* *HTSeq (v.0.6.1)[**87**]. Genes were retained if expressed in 80% of samples, with HTSeq quantification of 10 counts (thus removing genes supported by only a few reads) within all cortex and non-cortex samples separately. We used cqn software to adjust GC content according to the GC content results from the CollectGcBiasMetrics command in PicardTools as well as sequencing depth according to read length[**88**].*

*After that, we adjusted the data for covariates, including sex, batch, and sequencing quality metrics (Additional file 2, Table S1). Given the large number of sequencing quality features, we performed principal component analysis (PCA) on these data and found that the first two PCs on the unstandardized features explained nearly 99% of the variance. Consequently, we opted to use two sequencing surrogate variables (seqSV1 and seqSV2) as covariates. We applied a linear model to remove the confounding factors:*

*adjusted_value = original_value– batch*beta.batch- sex*beta.sex- seqSV1*beta.seqSV1 - seqSV1*beta.seqSV2.”*

-Statistics:
MWU tests are performed to compare DEG as well as the correlations between area and hemispheres but there is no mention of adjusted p-values for multiple testing in the methods. Adjustment is only described for the alternative splicing (DEU) analysis.

**Reply:**

**The *p*-values were adjusted in this study, which we have described in the revised manuscript.**

**Mann-Whitney U (MWU) test was performed by R function** **wilcox.test(), the command of correct = TRUE was used to adjust *p*-values, and a continuity correction was applied to the normal approximation for the *p*-values. Differentially expressed genes (DEGs) were analyzed using deseq2[10], and the *p-*value was adjusted for multiple testing using** [**Benjamini-Hochberg**](http://en.wikipedia.org/wiki/False_discovery_rate) **FDR. The analysis of alternative splicing (DEU) was performed by dexseq2[11], and the *p*-value was also adjusted for multiple testing using** [**Benjamini-Hochberg**](http://en.wikipedia.org/wiki/False_discovery_rate) **FDR.**

2. Results:
-Overall, the manuscript language is lacking precision; to the point that, at time, there seems to be overstated claims.
For example, the claim of alteration of the metabolic rate of the PGLS transduced mice where one would expect to find a difference in energy expanded over a certain period of time but only get a measure of food intake and dejection weights.

*Reply:*

*Thank you. According to your comment, we have narrowed down some claims. We agree that “overexpression of PGLS in mice induced alteration of metabolic rate” may be overstated. Metabolic rate is a complex process, which involves the measurement of multiple indicators. Measures of food intake and dejection weights are not enough to stand for metabolic rate.*

*We revised the description as follows:*

*“As aging usually accompanies a physiological decrease in food intake [67-70], we also tested food intake in the AAV-PGLS and Ctrl mice. Results showed that AAV-PGLS mice exhibited decreased food intake and fecal output compared to the Ctrl mice (Fig. 5j–k), indicating that overexpression of PGLS resulted in decreased appetite, a key indicator of aging[67].”*

The fact that PGLS overexpression induces synapses loss and increased apoptosis does not necessarily mean that synapses loss is caused by a higher rate of apoptosis.

*Reply:*

*Thank you. We agree with your statement. We have revised our description, and removed “synapses loss is caused by a higher rate of apoptosis”.*

The authors should clearly mention in the abstract and the discussion that PGLS validation was is done in the mouse.

*Reply:*

*Thank you very much for this advice. According to your suggestion, we clarified that PGLS validation was done in mice in the abstract and discussion.*

*In the abstract: “We further confirmed the functional significance of PGLS in mice at the gene transcription, molecular, and behavioral levels.”*

*In discussion: “Up-regulation of PGLS in mice resulted in impaired memory and locomotor activity, as well as decreased food intake”.*

-Figure1: The diagram nicely summarizes the strategy used in the manuscript. Maybe a separate diagram reporting the validation of PGLS in the mouse would make it even clearer.

There is no mention in the legend to the regions colored in green, blue, and red; what are they referring to?

*Reply:*

*Thank you. According to your suggestion, we revised Figure 1 and made a separate diagram reporting the functional validation of PGLS on brain aging in the mice.*

*There was no particular sense of different colors. To avoid this confusion, we only used one color to show 44 brain regions in the revision.*

**Fig. 1 | Schematic view of this study.** We used four young and three aged macaques across 44 brain regions to study aging mechanism in NHPs through multifaceted analyses (connectivity analysis, differentially expressed gene analysis, alternative splicing analysis, and network analysis). We further confirmed the role of *PGLS* underlying brain aging in mice. The table on the right show the ontology and nomenclature of analysed brain regions.

-Figure2: The heatmap matrices would be more informative and the age-dependent alterations more visible if the matrices reporting the inter areas and inter hemispheres correlations were built with the brain regions ordered the way in the young and old groups.

*Reply:*

*Thank you. According to your suggestion, we revised Figure 2 and ordered the correlation of brain regions in the same way for young and aged groups.*

**Fig. 2 | Expression connectivity between pairs of cerebral cortex areas and expression symmetry between left and right hemispheres decrease after aging. a** Heat map matrix of pairwise Pearson correlations between cortex regions (top) and between non-cortex areas (bottom) in young and aged macaques. **b** Heatmap matrix of pairwise Pearson correlations between left-right hemispheres in cortex (top) and non-cortex (bottom) regions in young and aged macaques (colnames represents brain areas across left hemisphere; rownames represents brain areas across right hemisphere).

-Figure3: (a) Is there some correlation between DEG and AS across brain regions?
(b, c, d) These 3 heatmaps are flattened by a few extreme p-values and consequently not very informative. For b and c, numbers or relative numbers of shared DEG between pair of regions would make the heatmaps more legible. Regarding (d), why not acorrelation plot?

(f) A few examples of genes associated with each cellular type and affected in a large number of regions would be more informative.

*Reply:*

*Thank you for your suggestions.*

*For Figure a, according to your suggestions, we now performed a correlation analysis, and found a positive correlation between the number of DEGs and gene with differential exon usage genes (DEUs) across brain regions (Pearson r2 = 0.39, Adjusted P value = 0.018). We have added this to the Supplementary Material.*

**Fig. S6.** Correlation plot of number of differentially expressed genes (DEGs) and genes with differential exon usage genes (DEUs) across brain regions (Pearson correlation test, a continuity correction is used for p value).

*For Figure b, c, d, according to your comment, we now used the ratio of intersection over union to show the relative numbers of shared DEGs or AS between pairs of brain regions. Regarding Figure d, we aimed to present that there are rare overlap between* *the gene sets of DEGs and genes with DEUs across pairwise brain regions, and we found that a heatmap may be better to display our results.*

**Fig. 3 | Aging-related transcriptional profile changes. a** The number of genes with evidence of aging-related gene expression (red) and aging-related alternative splicing (blue) changes. **b** The overlapping rate of DEGs between any two brain regions (The ratio of intersection over union was used to exhibit the overlapping rate). **c** The overlapping rate of genes with DEUs between any two brain regions. **d** The overlapping rate of DEUs and genes with DEGs between any two brain regions. **e** Enriched categories for up-regulated (top) and down-regulated (bottom) DEGs in aged macaques. **f** Matrix summary of enrichment in oligodendrocyte, neuron, microglia, endothelial, or astrocyte genes in up-regulated and down-regulated DEGs of aged macaques.

*For Figure f, we found up-regulated genes in aged monkey brains were enriched in astrocyte cells, whereas down-regulated genes in aged monkey brains were enriched in microglia cells. According to your suggestions, we have added a bar plot in the Supplementary Material to present a few examples of genes associated with astrocytes or microglia cells as well as differentially expressed between young and aged monkeys in a large number of brain regions.*

**Fig. S9.** Genes associated with astrocytes (upper) and microglia cells (below) that were differentially expressed in a large number of regions.

-Figure4: (f) The edges and the cyan dot are not visible.

*Reply:*

*Thank you. According to your comment, we revised Figure 4f for clarity.*

-Figure5: (i) Open field: the representative traces seem to show the inverse of the histograms.

*Reply:*

*Thank you for pointing out this error, which has now been corrected in the revised manuscript.*

(j) Food intake: on this panel PGLS mice are in black while they are in red in all other panels.

*Reply:*

*Thank you. We have revised Figure 5j.*

Minor comments:
1. Overall, the manuscript is still full of typos and misused words (for example several occurrences of "alternations" for "alterations" lines 57, 82…). It would greatly benefit from a native speaker editing

*Reply:*

*Thank you for your careful reading. We have thoroughly revised our manuscript with the assistance of a native English editor. We hope that all grammar/typo mistakes have been found and corrected in our paper.*

2. "from 3 young and 4 aged rhesus macaques" (line 80) instead of 4 young and 4 old macaques

*Reply:*

*Thank you. This has been revised.*

3. While the analysis of a published human dataset is interesting (lines127-133), a few descriptive sentences regarding the age range of the subjects and the brain areas sampled would help

*Reply:*

*Thank you. We have added a descriptive sentence regarding the age range and added a table to present human brain areas in the Supplementary Material to make our paper easier to follow.*

4. Mouse RNAseq experimental details and analysis are missing in the methods section.

*Reply:*

*Thank you. We have added a paragraph in the Method to describe Mouse RNAseq experimental details and analysis.*

5. A sentence about macaque life stages and life expectancy would help the reader to put the 2 experimental groups in context.

*Reply:*

*Thank you, we have added a description of macaque life stages and life expectancy in the revised manuscript.*

6. The figures resolution is too low and makes it difficult to evaluate.

*Reply:*

*We improved the figure resolution for clarity.*

7. What was the rational between choosing 6-month-old mice (at the beginning) instead of standard 2 months mice? One would expect an even greater contrast with younger adults.

*Reply:*

*We identified PGLS as a candidate gene of brain aging: i.e., overexpression of PGLS accelerated brain aging and induced aging-related phenotype. Standard 2-month-old mice are underage, thus using immature mice to overexpress PGLS will make the experiment cycle too long and the aging phenotype difficult to observe. However,* *6-month-old mice are young adults and considered mature, which is appropriate for studying the aging phenotype. And previous study also chose 6-month mice as experimental model to study a candidate gene (STEP) on brain aging[12]. Therefore, we think it is better to use 6-month-old mice to study brain aging.*

*REFERENCES*

*1.	Zerbino DR, Achuthan P, Akanni W, Amode M R, Barrell D, Bhai J, Billis K, Cummins C, Gall A, Girón CG, et al: Ensembl 2018. Nucleic Acids Research 2018, 46:D754-D761.*

*2.	Brawand D, Soumillon M, Necsulea A, Julien P, Csárdi G, Harrigan P, Weier M, Liechti A, Aximu-Petri A, Kircher M, et al: The evolution of gene expression levels in mammalian organs. In Nature, vol. 478. pp. 343-348; 2011:343-348.*

*3.	Zhang Y, Chen K, Sloan SA, Bennett ML, Scholze AR, Keeffe S, Phatnani HP, Guarnieri P, Caneda C, Ruderisch N, et al: An RNA-Sequencing Transcriptome and Splicing Database of Glia, Neurons, and Vascular Cells of the Cerebral Cortex. The Journal of Neuroscience 2014, 34:11929.*

*4.	Gray SJ, Foti SB, Schwartz JW, Bachaboina L, Taylor-Blake B, Coleman J, Ehlers MD, Zylka MJ, McCown TJ, Samulski RJ: Optimizing promoters for recombinant adeno-associated virus-mediated gene expression in the peripheral and central nervous system using self-complementary vectors. Human gene therapy 2011, 22:1143-1153.*

*5.	Galluzzi L, Vanpouille-Box C, Bakhoum SF, Demaria S: SnapShot: CGAS-STING Signaling. Cell 2018, 173:276-276 e271.*

*6.	Au - Young K, Au - Morrison H:* *Quantifying Microglia Morphology from Photomicrographs of Immunohistochemistry Prepared Tissue Using ImageJ. JoVE 2018:e57648.*

*7.	Scott JA, Grayson D, Fletcher E, Lee A, Bauman MD, Schumann CM, Buonocore MH, Amaral DG: Longitudinal analysis of the developing rhesus monkey brain using magnetic resonance imaging: birth to adulthood. Brain Struct Funct 2016, 221:2847-2871.*

*8.	Kang HJ, Kawasawa YI, Cheng F, Zhu Y, Xu X, Li M, Sousa AM, Pletikos M, Meyer KA, Sedmak G, et al: Spatio-temporal transcriptome of the human brain. Nature 2011, 478:483-489.*

*9.	Hawrylycz MJ, Lein ES, Guillozet-Bongaarts AL, Shen EH, Ng L, Miller JA, van de Lagemaat LN, Smith KA, Ebbert A, Riley ZL, et al: An anatomically comprehensive atlas of the adult human brain transcriptome. Nature 2012, 489:391-399.*

*10.*	*Love MI, Huber W, Anders S: Moderated estimation of fold change and dispersion for RNA-seq data with DESeq2. Genome Biology 2014, 15:550.*

*11. Anders S, Reyes A, Huber W: Detecting differential usage of exons from RNA-seq data. Genome research 2012, 22:2008-2017.*

*12.	Castonguay D, Dufort-Gervais J, Menard C, Chatterjee M, Quirion R, Bontempi B, Schneider JS, Arnsten AFT, Nairn AC, Norris CM, et al: The Tyrosine Phosphatase STEP Is Involved in Age-Related Memory Decline. Curr Biol 2018, 28:1079-1089 e1074.*

**Second round of review**

**Reviewer 1**

authors have thoroughly addressed my comments in the revised manuscript. now I would like to recommend this manuscript to be accepted  for publication in Genome Biology.

**Reviewer 2**

The authors did a great job revising their manuscript; they properly addressed my comments. The manuscript is now ready for publication.
